# Unassisted Clinicians Versus Deep Learning–Assisted Clinicians in Image-Based Cancer Diagnostics: Systematic Review With Meta-analysis

**DOI:** 10.2196/43832

**Published:** 2023-03-02

**Authors:** Peng Xue, Mingyu Si, Dongxu Qin, Bingrui Wei, Samuel Seery, Zichen Ye, Mingyang Chen, Sumeng Wang, Cheng Song, Bo Zhang, Ming Ding, Wenling Zhang, Anying Bai, Huijiao Yan, Le Dang, Yuqian Zhao, Remila Rezhake, Shaokai Zhang, Youlin Qiao, Yimin Qu, Yu Jiang

**Affiliations:** 1 Department of Epidemiology and Biostatistics School of Population Medicine and Public Health Chinese Academy of Medical Sciences and Peking Union Medical College Beijing China; 2 Faculty of Health and Medicine Division of Health Research Lancaster University Lancaster United Kingdom; 3 Department of Cancer Epidemiology National Cancer Center/National Clinical Research Center for Cancer/Cancer Hospital Chinese Academy of Medical Sciences and Peking Union Medical College Beijing China; 4 Peking Union Medical College Hospital Chinese Academy of Medical Sciences and Peking Union Medical College Beijing China; 5 Sichuan Cancer Hospital & Institute, Sichuan Cancer Center School of Medicine University of Electronic Science & Technology of China Sichuan China; 6 Affiliated Cancer Hospital The 3rd Affiliated Teaching Hospital of Xinjiang Medical University Xinjiang China; 7 Henan Cancer Hospital Affiliated Cancer Hospital of Zhengzhou University Henan China; 8 Center for Global Health School of Population Medicine and Public Health Chinese Academy of Medical Sciences and Peking Union Medical College Beijing China

**Keywords:** deep learning, cancer diagnosis, systematic review, meta-analysis

## Abstract

**Background:**

A number of publications have demonstrated that deep learning (DL) algorithms matched or outperformed clinicians in image-based cancer diagnostics, but these algorithms are frequently considered as opponents rather than partners. Despite the clinicians-in-the-loop DL approach having great potential, no study has systematically quantified the diagnostic accuracy of clinicians with and without the assistance of DL in image-based cancer identification.

**Objective:**

We systematically quantified the diagnostic accuracy of clinicians with and without the assistance of DL in image-based cancer identification.

**Methods:**

PubMed, Embase, IEEEXplore, and the Cochrane Library were searched for studies published between January 1, 2012, and December 7, 2021. Any type of study design was permitted that focused on comparing unassisted clinicians and DL-assisted clinicians in cancer identification using medical imaging. Studies using medical waveform-data graphics material and those investigating image segmentation rather than classification were excluded. Studies providing binary diagnostic accuracy data and contingency tables were included for further meta-analysis. Two subgroups were defined and analyzed, including cancer type and imaging modality.

**Results:**

In total, 9796 studies were identified, of which 48 were deemed eligible for systematic review. Twenty-five of these studies made comparisons between unassisted clinicians and DL-assisted clinicians and provided sufficient data for statistical synthesis. We found a pooled sensitivity of 83% (95% CI 80%-86%) for unassisted clinicians and 88% (95% CI 86%-90%) for DL-assisted clinicians. Pooled specificity was 86% (95% CI 83%-88%) for unassisted clinicians and 88% (95% CI 85%-90%) for DL-assisted clinicians. The pooled sensitivity and specificity values for DL-assisted clinicians were higher than for unassisted clinicians, at ratios of 1.07 (95% CI 1.05-1.09) and 1.03 (95% CI 1.02-1.05), respectively. Similar diagnostic performance by DL-assisted clinicians was also observed across the predefined subgroups.

**Conclusions:**

The diagnostic performance of DL-assisted clinicians appears better than unassisted clinicians in image-based cancer identification. However, caution should be exercised, because the evidence provided in the reviewed studies does not cover all the minutiae involved in real-world clinical practice. Combining qualitative insights from clinical practice with data-science approaches may improve DL-assisted practice, although further research is required.

**Trial Registration:**

PROSPERO CRD42021281372; https://www.crd.york.ac.uk/prospero/display_record.php?RecordID=281372

## Introduction

Cancer is a leading cause of death, with an estimated 19.3 million new cases and 10 million deaths in 2020 worldwide [1]. One of the reasons for this high burden is delayed diagnosis due to inconspicuous symptoms at an early stage [2]. Cancer identification is often only feasible once serious symptoms manifest or a lesion (or tumor) is large enough to be identified using conventional diagnostic imaging techniques [3]. State-of-the-art medical imaging technologies make early diagnosis possible and instill optimism; however, subjectivity in cancer imaging diagnosis influences the application of these technologies for individual patients. Of course, specialists tend to be more accurate, but such expertise is not widely available [4]. The emergence of deep learning (DL) algorithms in medical artificial intelligence (AI) provides a way forward, despite potentially causing disruptions to standardized, established practice [5].

As a subfield of AI, DL is formally defined as “computational models, composed of multiple processing layers, to learn representations of data with multiple levels of abstraction” [6]. In medical imaging practice, DL algorithms extract representative imaging features for classification purposes, irrespective of personal experience and underlying assumptions [7]. Over the past decade, we have witnessed a growing interest in DL algorithms, specifically in cancer diagnostics. Numerous studies have reported the diagnostic performance of DL, and it is considered comparable to, or in some circumstances better than, that of clinicians [8-10]. However, medical DL is plagued with issues, including inherent biases due to limited training data, an absence of cross-population generalizability, and a lack of transparency and accountability for clinical practice [11]. Therefore, the evidence required to change policy and implement DL techniques is insufficient. In fact, many appear preoccupied with the debate around medical AI replacing human physicians. However, these technologies are designed to assist clinicians and improve diagnostics within existing clinical workflows.

Human-computer collaboration can provide benefits above and beyond what either clinicians or DL algorithms can do in isolation [12-14]. This paradigm shift means that while the advantages of DL algorithms are necessary, so too are those of clinicians, who will need to fill gaps with personal knowledge of clinical histories. However, before implementing DL assistance into clinical practice, we must also assess current evidence in terms of methods and risk of bias. DL technologies must be subjected to the same rigorous assessment as any other technology in modern, evidence-based medicine. Doing anything else would impact patient acceptance and would impair the development of best practices for medical AI. There is also the wider issue of the impact on public trust in medicine, which cannot be overlooked. Therefore, it is imperative to systematically review the diagnostic performance of DL-assisted clinicians versus unassisted clinicians. Critically reviewing the evidence base is necessary to ensure these methods are both safe and effective. The synthesized evidence may also provide insights into the human factors involved in the use of DL assistance in cancer identification.

## Methods

### Search Strategy and Selection Criteria

The study protocol was registered with PROSPERO (CRD42021281372) and was conducted and reported in accordance with the PRISMA (Preferred Reporting Items for Systematic Reviews and Meta-analyses) 2020 guidelines [15].

Keywords, including “cancer,” “AI/DL,” “performance,” and “image” were used to identify comparative studies that assessed the performance of unassisted clinicians and DL-assisted clinicians in image-based cancer diagnosis. Multimedia Appendix 1 provides an outline of the full search strategy. Records from PubMed, Embase, IEEEXplore, and the Cochrane Library from January 1, 2012, to December 7, 2021, were systematically searched with no language restrictions. The start date was chosen based on a recognized step change in the development of DL approaches [16].

The inclusion and exclusion criteria for related studies were jointly determined by 2 independent authors who screened titles and abstracts. All studies were then read in full and discrepancies were resolved by a third author. Studies were included if they focused on comparing the performance of unassisted clinicians and DL-assisted clinicians in image-based cancer diagnosis. Studies were excluded if they (1) examined medical waveform-data graphics material, (2) investigated image segmentation rather than cancer identification, (3) reported ternary diagnostic outcomes, or (4) did not study DL. Case reports, reviews, editorials, letters, comments, conference abstracts or proceedings, and duplicates were also excluded (Multimedia Appendix 1).

### Data Extraction

Two authors independently extracted study characteristics, model-related details, and performance data. Uncertainties were resolved by another independent research associate. Binary diagnostic accuracy data, including true positives, false positives, true negatives, and false negatives, were extracted by 2 reviewers. Sensitivity and specificity data were then pooled for analysis. If a study provided a number of contingency tables for the same or different DL models, we assumed these were independent, unless otherwise stated.

### Quality Assessment

The Quality Assessment of Diagnostic Accuracy Studies 2 (QUADAS-2) tool was used to assess the risk of bias and applicability concerns of the included studies [17].

### Statistical Analysis

Hierarchical summary receiver operating characteristic (HSROC) curves were used to estimate overall accuracy. In the HSROC curves, 95% CIs and 95% prediction regions of the summary operating points, including averaged sensitivity, specificity, and the area under the curve (AUC), were provided under a random effects model. Heterogeneity was assessed using the *I*^2^ statistic.

Relative sensitivity and specificity were pooled for meta-analysis. Cancer type and imaging modality were established for subgroup analysis. Potential sources of heterogeneity were assessed across relative sensitivity and specificity for both DL-assisted clinicians and unassisted clinicians. Additional efforts were made to identify sources of heterogeneity in 2 separate subgroup meta-analyses: (1) according to cancer type, which included breast, lung, gastrointestinal, and endocrine cancers, and (2) according to imaging modality, which included ultrasound, X-ray, endoscopy, and magnetic resonance imaging (MRI).

The random effect model was implemented because of inherent differences within the evidence base. Publication bias was visually assessed using funnel plots. Only studies with N≥4 were included for statistical pooling. All analyses were conducted with Stata (version 15.1; Stata Corp) and SAS (version 9.4; SAS Institute). Two-sided *P* values of less than .05 were considered statistically significant.

## Results

### Study Selection

**Figure 1:**
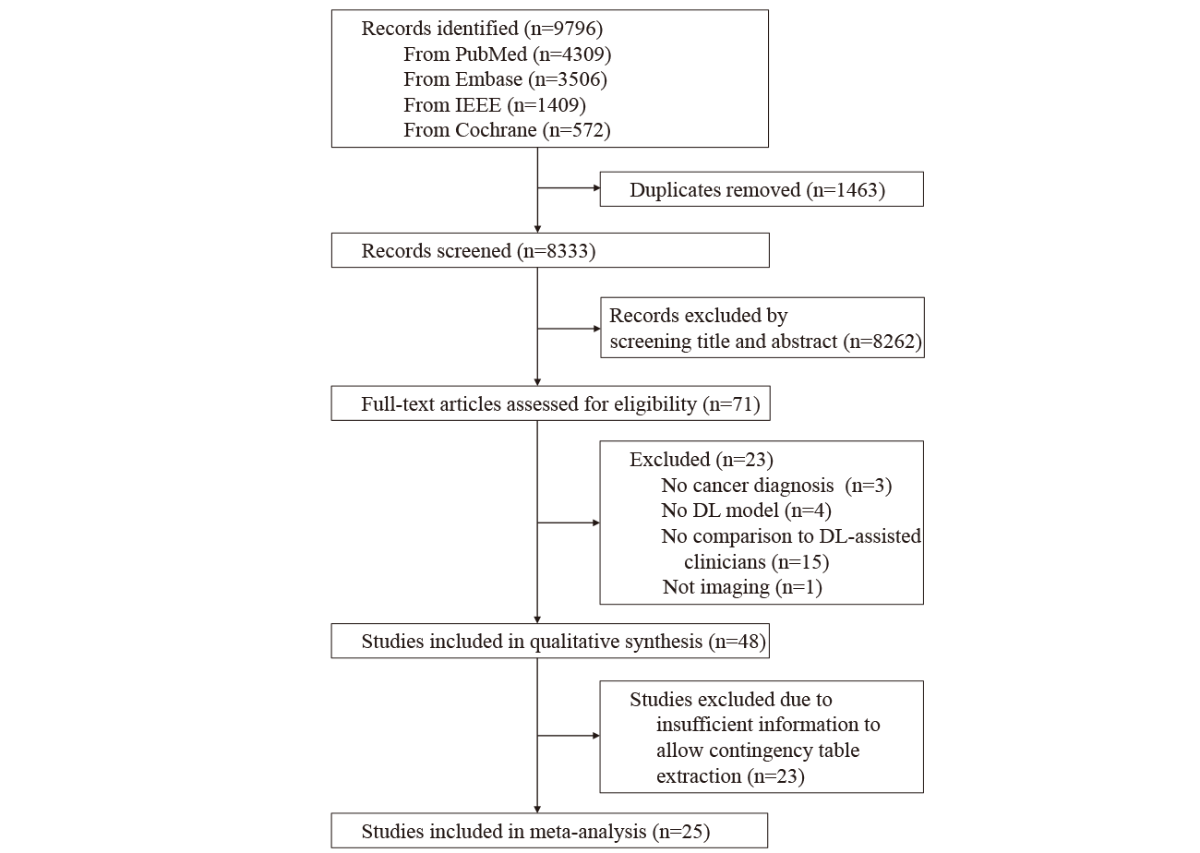
PRISMA (Preferred Reporting Items for Systematic Reviews and Meta-Analyses) flowchart of study selection. DL: deep learning.

Online searching was last updated on December 7, 2021, and 9796 studies were retrieved (Figure 1). After removing duplicates, 8333 publications were screened. After screening and selection, 71 full texts were considered eligible, although a further 23 were excluded due to insufficient information. This left 48 studies for systematic review.

### Characteristics of the Included Studies

Of the 48 enrolled studies [18-65], 52% (n=25) provided comprehensive inclusion and exclusion criteria while 12% (n=6) provided no information about participants (Multimedia Appendix 1). Breast and gastrointestinal cancer accounted for half the studies. The top 4 conditions were breast cancer (n=13, 27%), gastrointestinal cancer (n=11, 23%), lung cancer (n=8, 17%), and endocrine cancer (n=7, 15%). The top 4 imaging modalities were ultrasound (n=8, 17%), X-ray (n=8, 17%), and computerized tomography (n=7, 15%), with MRI and whole-slide imaging both used in 12% (n=6) of studies. Dermoscopy and endoscopy had 5 studies each, with each representing 10% of the sample. Multimedia Appendix 1 provides summaries of study characteristics. In total, 98% (n=47) of the studies were based on retrospectively collected data. Only 1 study could be considered prospective. Only 4 studies reported a prespecified sample size calculation. Meanwhile, 29% (n=14) of studies used data from open-access repositories. In addition, 54% (n=26) of studies performed external validation, whereas the remaining studies relied upon internal validation. Moreover, 33% (n=16) reported exclusion of low-quality images, and 35% (n=17) used heat maps. Transfer learning was applied in 21% of the studies (n=10) during the training phase.

Reference standards were wide ranging but in line with cancer types and imaging modalities, although some adopted multiple methods. Most of the studies (n=35, 73%) used histopathology with (or without) a follow-up period as the ground truth or gold standard control for image-based cancer diagnostics. The remainder implemented an expert consensus or medical record–based approach.

### Diagnostic Performance of Unassisted Clinicians Versus DL-Assisted Clinicians

**Figure 2:**
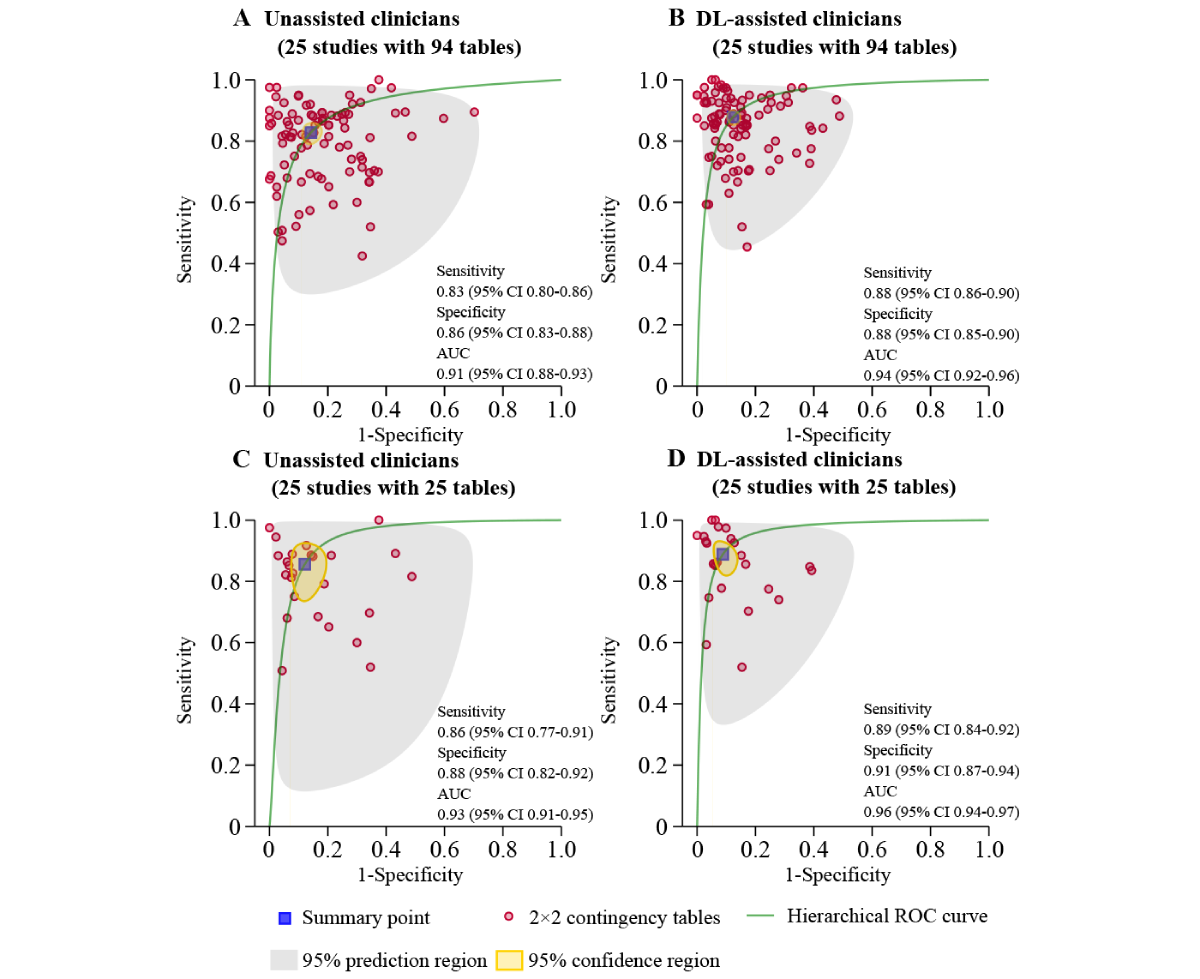
Hierarchical receiver operator characteristic curves of all studies included in the meta-analysis. A and B: ROC curves of all studies included in the meta-analysis (25 studies with 94 tables); C and D: ROC curves of studies reporting the highest accuracy (25 studies with 25 tables). AUC: area under the curve; DL: deep learning; ROC: receiver operator characteristic curve.

In total, 52% (n=25) of the included studies provided sufficient data to construct contingency tables, calculate diagnostic performance, and perform meta-analysis. HSROC curves generated using 25 studies (with a total of 94 contingency tables) are shown in Figure 2. When averaging across studies, the pooled sensitivity and specificity values for unassisted clinicians were 83% (95% CI 80%-86%) and 86% (95% CI 83%-88%), respectively, with an AUC of 0.91 (95% CI 0.88-0.93). By contrast, the pooled sensitivity and specificity values for DL-assisted clinicians were 88% (95% CI 86%-90%), and 88% (95% CI 85%-90%), respectively, with an AUC of 0.94 (95% CI 0.92-0.96). The pooled sensitivity and specificity values for DL-assisted clinicians were higher than those for unassisted clinicians at ratios of 1.07 (95% CI 1.05-1.09) and 1.03 (95% CI 1.02-1.05), respectively.

Most studies reported more than one DL algorithm for assessing diagnostic performance, and only the highest performance in each study was chosen for 25 contingency tables. The pooled sensitivity was 86% (95% CI 77%-91%) for unassisted clinicians and 89% (95% CI 84%-92%) for DL-assisted clinicians. The pooled specificity was 88% (95% CI 82%-92%) for unassisted clinicians and 91% (95% CI 87%-94%) for DL-assisted clinicians. The clustered AUCs for unassisted and DL-assisted clinicians were 0.93 (95% CI 0.91-0.95) and 0.96 (95% CI 0.94-0.97), respectively (Figure 2). The pooled sensitivity and specificity values for DL-assisted clinicians were higher than those for unassisted clinicians at ratios of 1.07 (95% CI 1.03-1.10) and 1.03 (95% CI 1.01-1.06), respectively.

### Subgroup Meta-analyses Comparing Diagnostic Performance

#### Cancer Type

**Figure 3:**
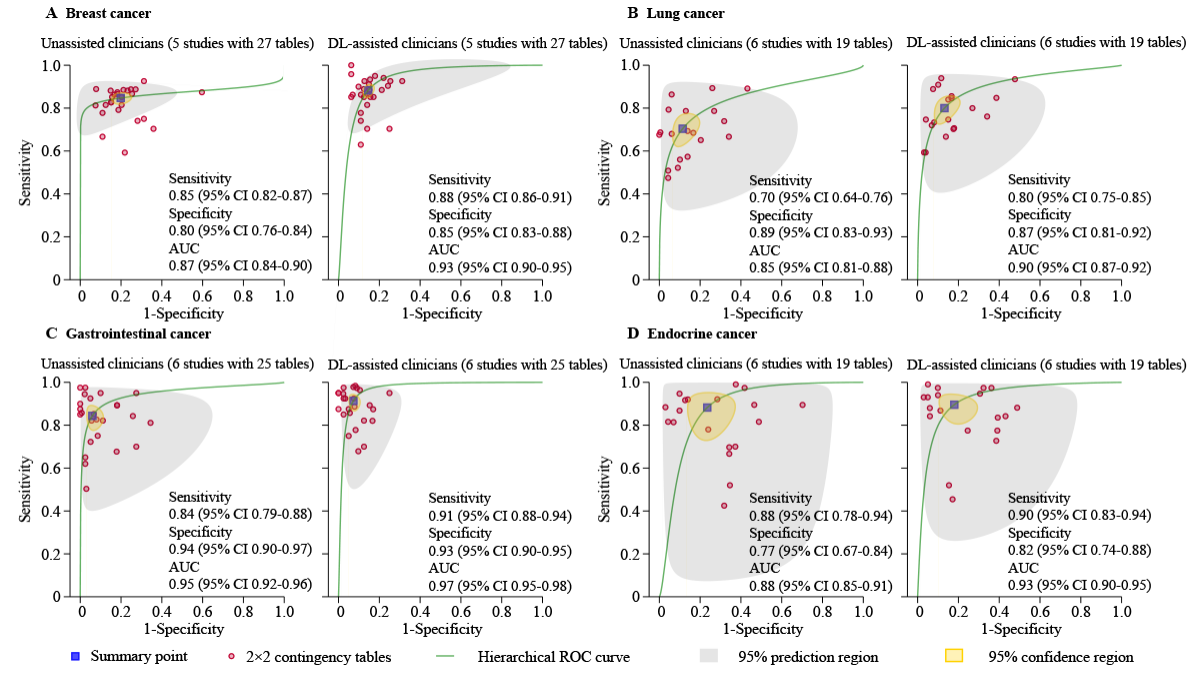
Hierarchical ROC curves of studies using different cancer types for comparing the performance of unassisted clinicians and DL-assisted clinicians. A: ROC curves of studies for detecting breast cancer (5 studies with 27 tables); B: ROC curves of studies for detecting lung cancer (6 studies with 19 tables); C: ROC curves of studies for detecting gastrointestinal cancer (6 studies with 25 tables); D: ROC curves of studies for detecting endocrine cancer (6 studies with 19 tables). AUC: area under the curve; DL: deep learning; ROC: receiver operator characteristic curve.

Five studies were used to create 27 contingency tables for breast cancer. The pooled sensitivity was 85% (95% CI 82%-87%) and specificity was 80% (95% CI 76%-84%), with an AUC of 0.87 (95% CI 0.84-0.90) for unassisted clinicians. For DL-assisted clinicians, we found a pooled sensitivity of 88% (95% CI 86%-91%) and specificity of 85% (95% CI 83%-88%), with an AUC of 0.93 (95% CI 0.90-0.95) (Figure 3A).

Six studies were used to develop 19 contingency tables for lung cancer. The pooled sensitivity was 70% (95% CI 64%-76%) for unassisted clinicians and 80% (95% CI 75%-85%) for DL-assisted clinicians. Pooled specificity was 89% (95% CI 83%-93%) for unassisted clinicians and 87% (95% CI 81%-92%) for DL-assisted clinicians, with an AUC of 0.85 (95% CI 0.81-0.88) for unassisted clinicians and 0.90 (95% CI 0.87-0.92) for DL-assisted clinicians (Figure 3B).

Six studies were used to generate 25 contingency tables for gastrointestinal cancer. The pooled sensitivity was 84% (95% CI 79%-88%), with a specificity of 94% (95% CI 90%-97%) and an AUC of 0.95 (95% CI 0.92-0.96) for unassisted clinicians. Pooled sensitivity was 91% (95% CI 88%-94%) and specificity was 93% (95% CI 90%-95%) with an AUC of 0.97 (95% CI 0.95-0.98) for DL-assisted clinicians (Figure 3C).

Another six studies were used to generate 19 tables for endocrine cancer. The pooled sensitivity was 88% (95% CI 78%-94%) for unassisted clinicians and 90% (95% CI 83%-94%) for DL-assisted clinicians. The pooled specificity was 77% (95% CI 67%-84%) for unassisted clinicians and 82% (95% CI 74%-88%) for DL-assisted clinicians, with an AUC of 0.88 (95% CI 0.85-0.91) for unassisted clinicians and 0.93 (95% CI 0.90-0.95) for DL-assisted clinicians (Figure 3D).

#### Imaging Modalities

**Figure 4:**
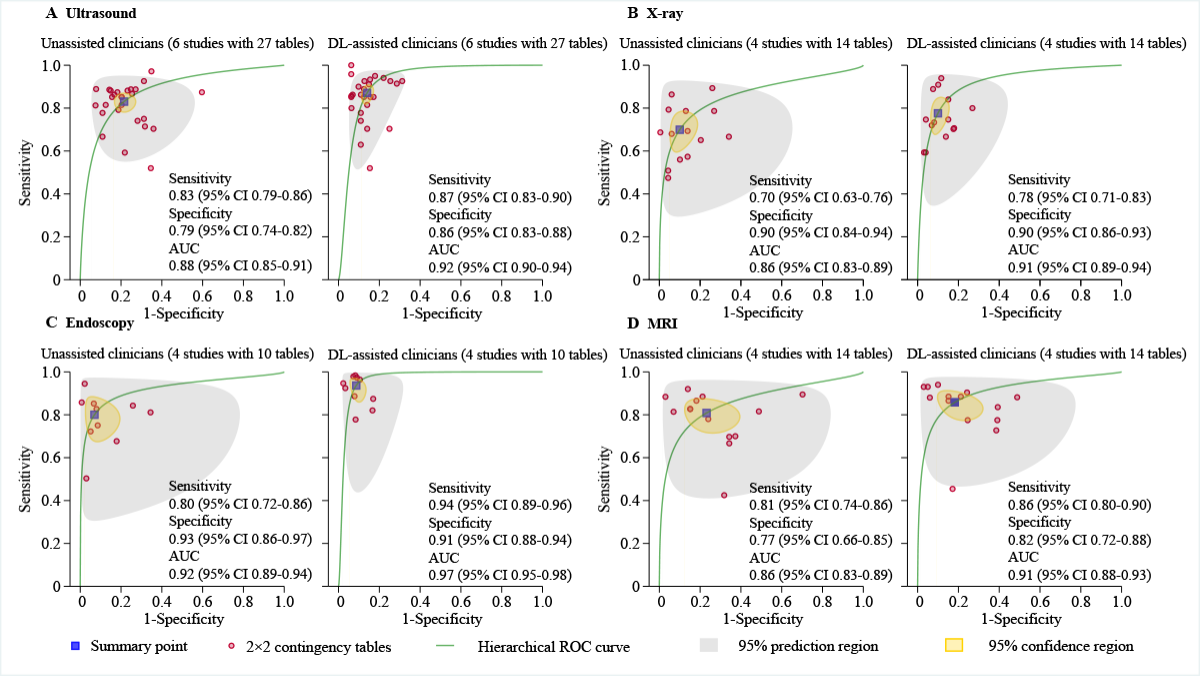
Hierarchical ROC curves of studies using different imaging modalities for comparing performance between unassisted clinicians and DL-assisted clinicians. A: ROC curves of studies using ultrasound (6 studies with 27 tables); B: ROC curves of studies using X-rays (4 studies with 14 tables); C: ROC curves of studies using endoscopy (4 studies with 10 tables); D: ROC curves of studies using MRI (4 studies with 14 tables). AUC: area under the curve; DL: deep learning; MRI: magnetic resonance imaging; ROC: receiver operator characteristic curve.

Six studies were used to generate 27 tables for ultrasound, which displayed a pooled sensitivity of 83% (95% CI 79%-86%) and specificity of 79% (95% CI 74%-82%), with an AUC of 0.88 (95% CI 0.85-0.91), for unassisted clinicians; the pooled sensitivity was 87% (95% CI 83%-90%) and specificity was 86% (95% CI 83%-88%), with an AUC of 0.92 (95% CI 0.90-0.94), for DL-assisted clinicians (Figure 4A).

For 4 X-ray studies, 14 tables were generated, which revealed a pooled sensitivity of 70% (95% CI 63%-76%) and specificity of 90% (95% CI 84%-94%), with an AUC of 0.86 (95% CI 0.83-0.89), for unassisted clinicians. Pooled sensitivity was 78% (95% CI 71%-83%) and specificity was 90% (95% CI 86%-93%), with an AUC of 0.91 (95% CI 0.89-0.94), for DL-assisted clinicians (Figure 4B).

Four endoscopy studies were used to create 10 tables that highlighted a pooled sensitivity of 80% (95% CI 72%-86%) for unassisted clinicians and 94% (95% CI 89%-96%) for DL-assisted clinicians. Pooled specificity was 93% (95% CI 86%-97%) for unassisted clinicians and 91% (95% CI 88%-94%) for DL-assisted clinicians, with AUCs of 0.92 (95% CI 0.89-0.94) for unassisted clinicians and 0.97 (95% CI 0.95-0.98) for DL-assisted clinicians (Figure 4C).

Additionally, there were 4 studies with 14 tables using MRI. The pooled sensitivity was 81% (95% CI 74%-86%) for unassisted clinicians and 86% (95% CI 80%-90%) for DL-assisted clinicians. Pooled specificity was 77% (95% CI 66%-85%) for unassisted clinicians and 82% (95% CI 72%-88%) for DL-assisted clinicians, with an AUC of 0.86 (95% CI 0.83-0.89) for unassisted clinicians and 0.91 (95% CI 0.88-0.93) for DL-assisted clinicians (Figure 4D). Detailed comparisons of subgroup meta-analyses (for cancer type and image modality) of the relative sensitivity and specificity of DL-assisted clinicians versus unassisted clinicians are shown in Table 1.

**Table 1:** Meta-analyses of the relative sensitivity and specificity of deep learning–assisted clinicians versus unassisted clinicians. *P* values represent a statistically significantly difference from unity (1 excluded from 95% CI, *P*<.05).

Deep learning–assisted clinicians versus unassisted clinicians		Studies, n	Tables, n	Relative sensitivity (95% CI)	*P* value	Relative specificity (95% CI)	*P* value
Overall		25	94	1.07 (1.05-1.09)	<.001	1.03 (1.02-1.05)	<.001
Studies reporting the highest performance^a^		25	25	1.07 (1.03-1.10)	<.001	1.03 (1.01-1.06)	.02
**Cancer type**							
	Breast cancer	5	27	1.05 (1.02-1.08)	<.001	1.08 (1.03-1.13)	.003
	Lung cancer	6	19	1.13 (1.08-1.18)	<.001	1.01 (0.99-1.03)	.36
	Gastrointestinal cancer	6	25	1.09 (1.04-1.14)	<.001	1.02 (0.99-1.04)	.26
	Endocrine cancer	6	19	1.01 (0.99-1.03)	.35	1.05 (1.01-1.09)	.02
**Imaging modality**							
	Ultrasound	6	27	1.04 (1.02-1.07)	<.001	1.10 (1.05-1.16)	<.001
	X-ray	4	14	1.11 (1.06-1.17)	<.001	1.01 (0.99-1.04)	.20
	Endoscopy	4	10	1.17 (1.09-1.25)	<.001	1.02 (0.99-1.06)	.27
	Magnetic resonance imaging	4	14	1.04 (1.00-1.09)	.08	1.06 (1.00-1.12)	.07

^a^Most studies reported more than one deep learning algorithm to assess diagnostic performance; only the highest-performing algorithm in each study was chosen for the 25 contingency tables.

### Heterogeneity Analysis

**Figure 5:**
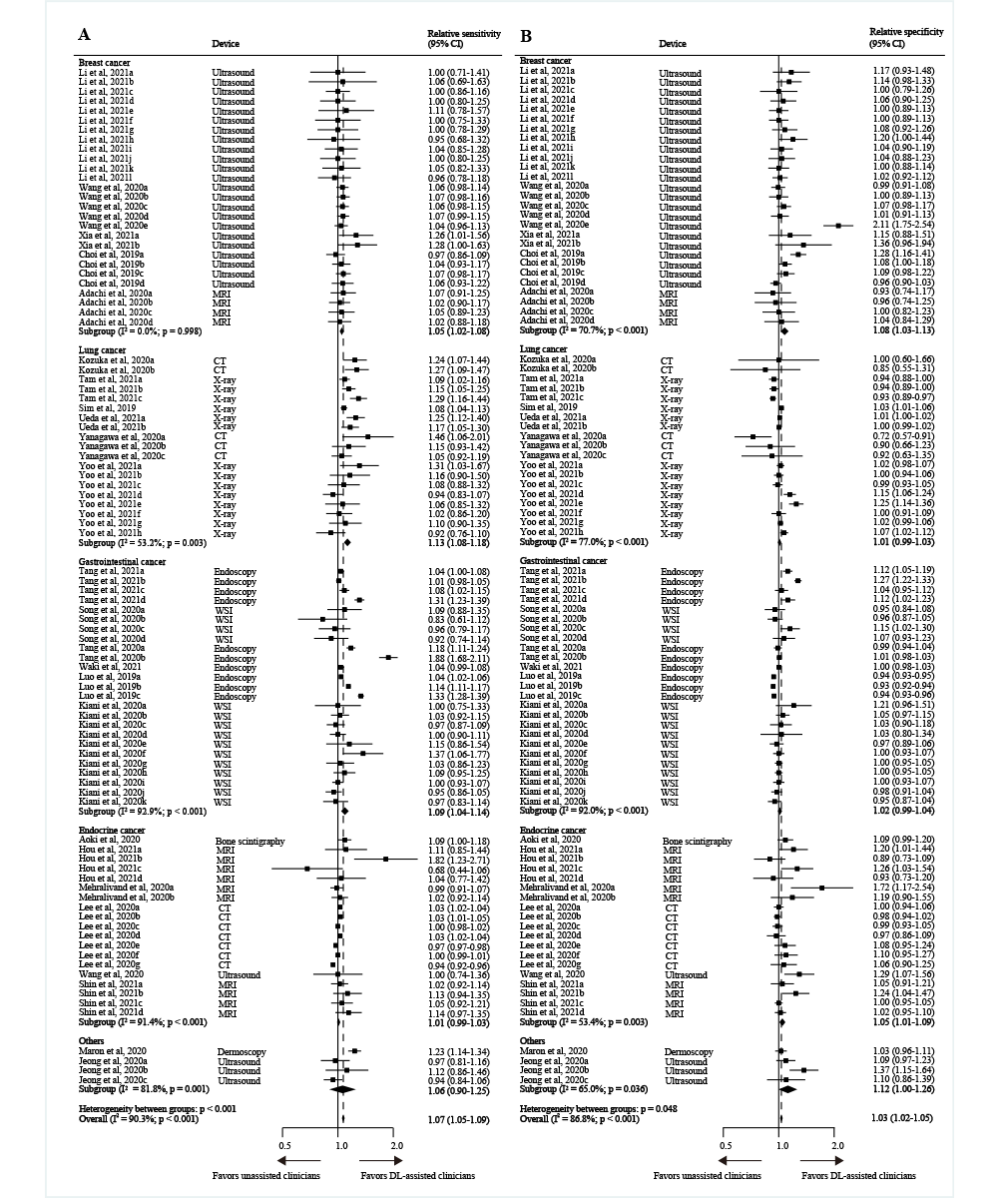
Pooled relative sensitivity (A) and specificity (B) of DL-assisted clinicians versus unassisted clinicians for different imaging modalities in image-based cancer detection. The data are presented as forest plots for all studies with different imaging modalities included in the meta-analysis (n=25 studies). If a study provided multiple contingency tables for DL-assisted clinicians versus unassisted clinicians, it is listed more than once and labelled alphabetically. The data are presented as forest plots for all studies with imaging modalities included in the meta-analysis (n=25 studies). CT: computed tomography; DL: deep learning; MRI: magnetic resonance imaging; ROC: receiver operator characteristic curve; WSI: whole-slide imaging.

**Figure 6:**
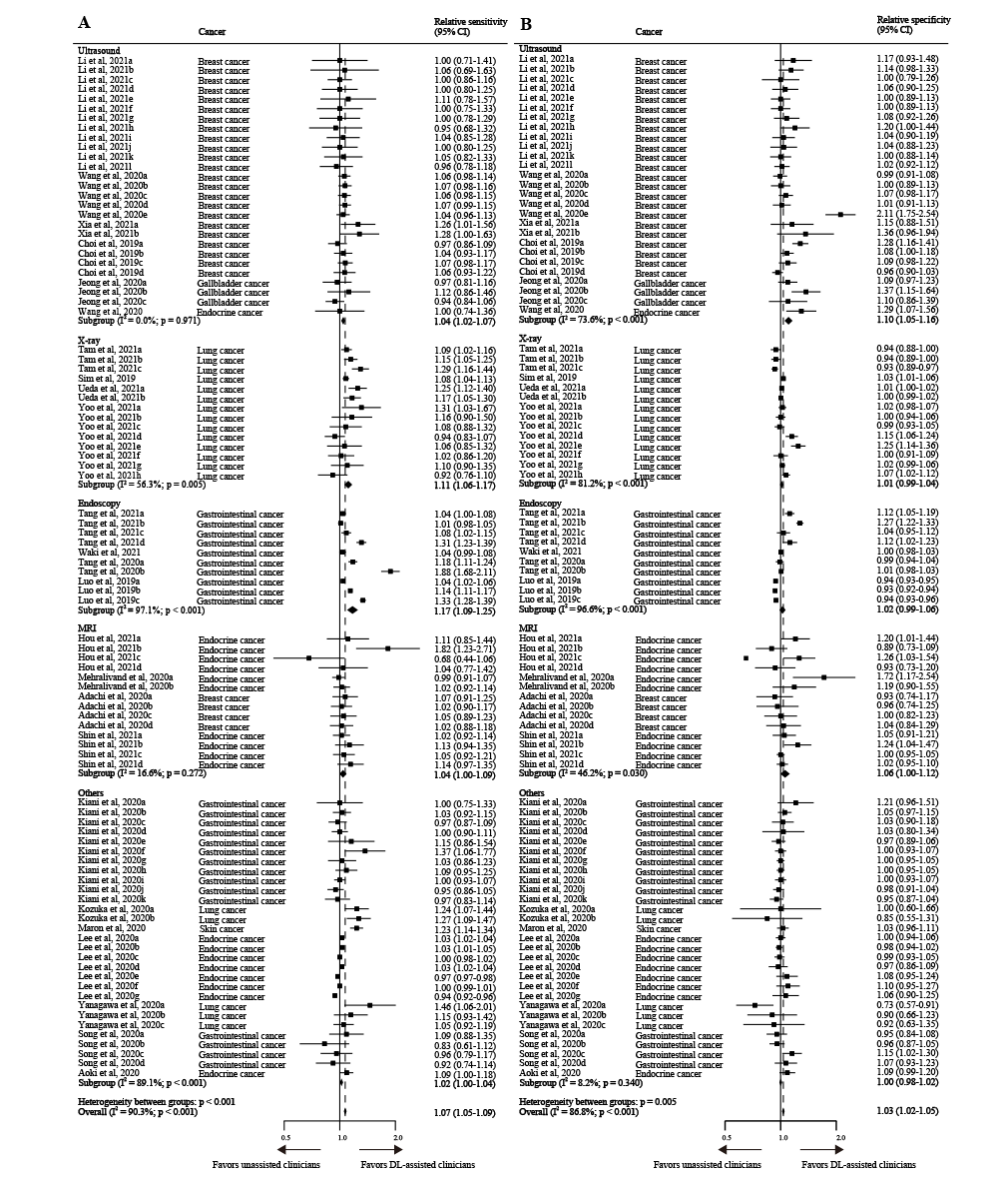
Pooled relative sensitivity (A) and specificity (B) of DL-assisted clinicians versus unassisted clinicians for different cancer types in image-based cancer detection. The data are presented as forest plots for all studies with different cancer types included in the meta-analysis (n=25 studies). If a study provided multiple contingency tables for DL-assisted clinicians versus unassisted clinicians, it is listed more than once and labelled alphabetically. DL: deep learning.

All included studies found that DL-assisted clinicians’ diagnostic performance appeared to be better than unassisted clinicians at image-based cancer identification; however, extreme heterogeneity was observed (*I*^2^ for sensitivity was 90.3%, *I*^2^ for specificity was 86.8%, *P*<.001) (Figures 5 and 6). This is discussed in more detail in the Limitations section.

A funnel plot was generated to assess publication bias. The studies appeared symmetrically distributed around the regression line, and a *P* value of .14 suggests no publication bias (Multimedia Appendix 1). To identify the source or sources of heterogeneity, we conducted a subgroup analysis. Although the heterogeneity for both sensitivity and specificity within several subgroups decreased to an acceptable range after grouping, the *I*^2^ values targeting overall diagnostic performance were still unsatisfactory. Therefore, cancer types and imaging modalities are likely to have confounded the image-based cancer diagnostic performance of unassisted clinicians versus DL-assisted clinicians.

### Quality Assessment

Quality was assessed using the QUADAS-2 tool; findings are summarized in Multimedia Appendix 1. The risk of bias and concerns of applicability for each study are also outlined in Multimedia Appendix 1. Nine and 25 studies were considered to have high and unclear risk in the patient-selection domain, respectively. Selection criteria in these studies were unreported, unclear, or considered inappropriate. The overall methodological quality was fair, and the applicability concerns were deemed acceptable.

## Discussion

### Principal Findings

We performed the first reported systematic review with a meta-analysis to assess the diagnostic accuracy of unassisted clinicians versus DL-assisted clinicians across distinct cancer types and imaging modalities. Evidence suggests that DL-assisted clinicians perform better at cancer identification than unassisted clinicians. DL-assisted clinicians also appeared to be superior across all cancer types and imaging modalities analyzed here. This suggests that DL assistance can be applied across different fields of image-based cancer identification.

Overall, the pooled sensitivity and pooled specificity values for DL-assisted clinicians were higher than for unassisted clinicians, at ratios of 1.07 (95% CI 1.05-1.09) and 1.03 (95% CI 1.02-1.05), respectively. Meta-analytical findings also support Budd et al [66] and Maadi et al [67], who acknowledged that a practical collaboration between humans and AI would improve clinical practice. Evidence is continually emerging that DL-assisted clinicians outperform unassisted clinicians in the diagnosis of breast and endocrine cancer, in terms of both sensitivity and specificity. Similar superiority occurs when using ultrasound. These results are consistent with previous research [68,69] that reported improvements in diagnostic performance. As for endocrine cancer, increments in specificity have been observed from 77% to 82%, despite there being no significant increases in sensitivity. Conversely, in lung and gastrointestinal cancer, increments in sensitivity have been observed from 70% to 80%, and from 84% to 91%, respectively, despite there being no significant increases in specificity. Similarly, when analyzing X-rays and endoscopic findings, increments in sensitivity have also been observed from 70% to 78% and from 80% to 94%, respectively, but there was no significant increase in specificity. The absence of a significant increase in specificity with DL assistance might be the result of a threshold effect. In other words, the lack of an improvement in specificity may have been a trade-off against an improvement in sensitivity. Among cancer types and imaging modalities, we observed that the sensitivity of DL-assisted clinicians ranged from 80% for lung cancer to 91% for gastrointestinal cancer, and from 78% with X-rays to 94% with endoscopy. The specificity of DL-assisted clinicians ranged from 82% for endocrine cancer to 93% for gastrointestinal cancer, and from 82% using MRI to 91% using endoscopy. Diagnostic performance disparities may be attributable to the participant composition of studies, designs, disease prevalence, clinical end points, cancer stage or histology type, and device type. This helps us to understand diagnostic gaps and to promote more accurate image-based cancer diagnoses.

### Analysis of the Main Aspects

These observations suggest more balanced cutoff points may be necessary to train DL models to augment diagnostic sensitivity. This may also mean that DL assistance should be matched with an accepted level of diagnostic specificity that has yet to be determined. Future work may look to focus on reducing the number of false-positive results, but there does appear to be a reduction in the number of false negatives that can be attributed to DL assistance. Another possibility for improving specificity while retaining high sensitivity might be to combine DL assistance models with advanced screening or diagnostic technologies. However, this will require a more detailed health-economics analysis, and regulatory bodies will need to consider the affordability of these new workflows. From a system perspective, DL assistance will have to be adapted to new, more advanced technologies while being trained to adapt to changing workflows, similar to human physicians.

Our findings support the notion that human-computer collaboration represents an improvement over (or is at least equivalent to) clinicians working without assistance to identify cancer cases. However, the knowledge base suffers from broad methodological deficiencies and poor reporting, although this could be overcome during training. This review shows that research in this area is still in the early stages of development. Less than half of the included studies were eligible for meta-analysis. Many studies were excluded at the initial screening stage because they only assessed the diagnostic performance of human intelligence versus machine intelligence, rather than human intelligence with DL assistance, which does not reflect a logical progression. Nevertheless, we acknowledge that assessing the accuracy of DL algorithms in isolation compared to human clinicians is often the first step for new technologies, although this does not represent a real-world situation, and conducting such polarized research may be the cause of anxieties within the profession. We hold the opinion that technologies are created to assist medical professionals, rather than being developed as replacements. Misunderstandings should be avoided and research should aim for human-in-the-loop DL for optimal integration. Therefore, we should work to integrate data science training into clinical training (and vice versa) to ensure research, at a fundamental level, is truly interdisciplinary and practicable.

While it is encouraging to see that DL assistance improves cancer case identification, caution should be exercised when applying our findings to clinical practice, because the studies under review were generally based upon in silico research. Reporting standards, which are essential to assess study quality, may not be considered as important by data scientists. There are certainly divergences in how research is conducted and reported that have impacted this emerging evidence base. In clinical research, comparative studies should be considered for the primary technical assessment of DL-assisted clinicians. This is not just about the quantification of diagnostic effects, as if it were, we would overestimate the benefit by overfitting. Of course, in silico research will continue to play an important role in simulating DL-assisted clinical practice and is useful for ensuring safety, effectiveness, and patient acceptability. However, these studies must be used to pave the way for large-scale clinical trials and then on to real-world studies. There are many gaps in this knowledge base, and as we anticipate a great deal of future research, the architecture of the knowledge base should be considered in more detail. Implementing DL assistance in clinical settings will require a blend of research methods, overlapping disciplines, and more sophisticated collaboration. This has implications for project and program leadership, although these topics are beyond the scope of this study. Suffice to say, if we are going to advance clinical practice, we ought to design DL assistance with reflective clinical advice and through mixed methods analysis, which demands improved reporting.

Recently, the DECIDE-AI (Developmental and Exploratory Clinical Investigations of Decision Support Systems Driven by Artificial Intelligence) reporting guidelines for the early-stage clinical evaluation of decision support systems were published [70]. The guidelines address issues in AI and DL development through exploratory research before large-scale efficacy testing is conducted. The majority of the studies included in this paper were probably conceived (and performed) before the DECIDE-AI guidelines were published. Therefore, it is reasonable to assume that the design features and reporting used to assess the diagnostic performance of DL-assisted clinicians will improve, as will transparency. However, with the future in mind, we should also assume that the DECIDE-AI guidelines are a prototype and will continue to be developed. Researchers will be at fault if they adopt these parameters without considering clinicians’ perspectives. Some have already commented that the use of human-in-the-loop AI or DL to support image-based cancer diagnostics might represent the optimal strategy for real-world clinical practice [66,67,71]. Nevertheless, we need to acknowledge that this is a rapidly evolving research area that will require subsequent updates. We are seeing the emergence of decentralized and hybridized trials that will, like interdisciplinary medical-AI research, need to accept increasingly sophisticated reporting standards.

Another major problem we encountered when considering discussions within each study was that of “cooperation.” Several researchers and theorists have already commented on the problems with possible cooperation modes between clinicians and DL algorithms [72]. For example, junior clinicians, who lack practical experience, may overly rely on AI while more experienced clinicians may find it difficult to make judgments and may find DL assistance forces them into a prolonged state of cognitive dissonance. This may also lead experienced experts and senior clinicians to distrust DL assistance, which may (in the long run) mean they are more likely to reject DL assistance. One possible approach to improving human-computer collaboration would be to leverage the advantages of DL algorithms (eg, rapid, automated detection) while having clinicians situated at various “checkpoints” to fill gaps where algorithms are not assured, or where they may fall short due to underlying biases. Cases are likely to vary, some with high confidence or, more concisely, with a high probability of the presence (or absence) of cancer relative to the probabilistic threshold for cancer detection, while others will be associated with less confidence. In cases with less confidence, outputs could be considered more closely by clinicians to generate combined decisions. This means we may need to weight DL-based decisions around algorithmic confidence, which may encourage symbiosis between human clinicians and DL algorithms.

We also note that approximately one-third of the included studies used data from open-access repositories, while the remainder made use of nonpublic data sets. These sources do not provide a valid within-source benchmark for comparison. Researchers have referred to the limited availability of open-access data and codes and the risk of bias and overfitting in existing DL research [9,73-75]. Our review supports these assertions and echoes these concerns. Lacking public data sets is a fundamental cause of the growing digital health divide [76]. Therefore, we encourage the DL and health care communities to collaborate and increase the number of studies that compare DL-assisted clinicians and unassisted clinicians. This will ensure clarity when designing interdisciplinary research and interpreting data, and it will also encourage acceptance by both clinicians and patients.

### Limitations and Recommendations

Before providing recommendations, we should discuss the limitations of this study. Our search strategy might have unintentionally excluded some pertinent DL-assisted studies and potentially useful non-English references. There were also substantial differences in patient characteristics, cancer types, imaging modalities, diagnostic thresholds, DL algorithms, and clinician experiences. These likely impacted measures of heterogeneity, although the fundamental purpose of this study was to systematically review evidence. We focused specifically on studies of DL-assisted clinicians and image-based cancer diagnostics. Therefore, the goal was not really to obtain generalizable findings but to identify gaps in our current knowledge. While we provide a quality assessment for transparency, the QUADAS-2 tool is suboptimal for assessing AI diagnostic research. Given the importance of AI and DL technologies, we would encourage global stakeholders to develop a new QUADAS-AI/DL tool that assesses the risk of bias and applicability.

Finally, we must emphasize that reliable estimates for performance can only be achieved through well-designed and well-executed studies that minimize bias in conduct and reporting. There remains uncertainty around the estimates of diagnostic performance provided in this exploratory meta-analysis, which should be considered before implementation.

### Conclusions

Human-machine cooperation in cancer diagnosis using medical imaging holds enormous potential. We found that the diagnostic accuracy of DL-assisted clinicians appeared better than unassisted clinicians. This area warrants further investigation, and we acknowledge that more rigorously designed, transparently reported, higher-quality studies are needed. This may help facilitate the transition of DL assistance into clinical practice, although further interdisciplinary mixed methods research is required.

## Appendix

Multimedia Appendix 1 Supplement document.

## Abbreviations

AI: artificial intelligence
AUC: area under the curve
DECIDE-AI: Developmental and Exploratory Clinical Investigations of Decision Support Systems Driven by Artificial Intelligence
DL: deep learning
HSROC: hierarchical summary receiver operating characteristic
MRI: magnetic resonance imaging
QUADAS-2: Quality Assessment of Diagnostic Accuracy Studies 2
