# Correction: Unassisted Clinicians Versus Deep Learning–Assisted Clinicians in Image-Based Cancer Diagnostics: Systematic Review With Meta-analysis

**DOI:** 10.2196/49146

**Published:** 2023-06-02

**Authors:** Peng Xue, Mingyu Si, Dongxu Qin, Bingrui Wei, Samuel Seery, Zichen Ye, Mingyang Chen, Sumeng Wang, Cheng Song, Bo Zhang, Ming Ding, Wenling Zhang, Anying Bai, Huijiao Yan, Le Dang, Yuqian Zhao, Remila Rezhake, Shaokai Zhang, Youlin Qiao, Yimin Qu, Yu Jiang

**Affiliations:** 1 Department of Epidemiology and Biostatistics School of Population Medicine and Public Health Chinese Academy of Medical Sciences and Peking Union Medical College Beijing China; 2 Faculty of Health and Medicine Division of Health Research Lancaster University Lancaster United Kingdom; 3 Department of Cancer Epidemiology National Cancer Center/National Clinical Research Center for Cancer/Cancer Hospital Chinese Academy of Medical Sciences and Peking Union Medical College Beijing China; 4 Peking Union Medical College Hospital Chinese Academy of Medical Sciences and Peking Union Medical College Beijing China; 5 Sichuan Cancer Hospital & Institute, Sichuan Cancer Center School of Medicine University of Electronic Science & Technology of China Sichuan China; 6 Affiliated Cancer Hospital The 3rd Affiliated Teaching Hospital of Xinjiang Medical University Xinjiang China; 7 Henan Cancer Hospital Affiliated Cancer Hospital of Zhengzhou University Henan China; 8 Center for Global Health School of Population Medicine and Public Health Chinese Academy of Medical Sciences and Peking Union Medical College Beijing China

In “Unassisted Clinicians Versus Deep Learning–Assisted Clinicians in Image-Based Cancer Diagnostics: Systematic Review With Meta-analysis” (J Med Internet Res 2023;25:e43832), the authors noted one error.

In the originally published article, the highest academic degrees for thirteen of the authors were erroneously displayed in the authorship list as follows:

Peng Xue, PhD; Mingyu Si, PhD; Dongxu Qin, MD; Bingrui Wei, MD; Zichen Ye, MD; Mingyang Chen, PhD; Sumeng Wang, PhD; Cheng Song, PhD; Bo Zhang, MD; Ming Ding, MD; Wenling Zhang, MD; Anying Bai, PhD; Huijiao Yan, PhD.

These have been corrected to:

Peng Xue, MPH; Mingyu Si, MPH; Dongxu Qin, BM; Bingrui Wei, BS; Zichen Ye, BM; Mingyang Chen, BM; Sumeng Wang, BM; Cheng Song, MPhil; Bo Zhang, BM; Ming Ding, BM; Wenling Zhang, BM; Anying Bai, BM; Huijiao Yan, MPH.

The correction will appear in the online version of the paper on the JMIR Publications website on June 2, 2023, together with the publication of this correction notice. Because this was made after submission to PubMed, PubMed Central, and other full-text repositories, the corrected article has also been resubmitted to those repositories.

