# Supplementary material for: Unassisted Clinicians Versus Deep Learning–Assisted Clinicians in Image-Based Cancer Diagnostics: Systematic Review With Meta-analysis
Source: J Med Internet Res. 2023 Mar 2;25:e43832. doi: 10.2196/43832 (PMC10020907; doi:10.2196/43832)
Supplement: Multimedia Appendix 1 [file jmir_v25i1e43832_app1.docx]

**Supplement Document**

**Clinicians versus deep learning-assisted clinicians for** **image-based cancer diagnostics: a systematic review with meta-analysis**

**Search terms and search strategy**

**1) PubMed search strategy:** 4309

#1 "artificial intelligence"[Mesh] OR "machine learning"[Mesh] OR "deep learning"[Mesh] OR "neural network, computer"[Mesh]

#2 (artificial intelligence*[Title/Abstract]) OR (machine learning*[Title/Abstract]) OR (deep learning*[Title/Abstract]) OR (neural network*[Title/Abstract])

#3 #1 OR #2

#4 "random forest"[Mesh] OR "decision trees"[Mesh] OR "support vector machine"[Mesh]

#5 (decision tree*[Title/Abstract]) OR (random forest[Title/Abstract]) OR (nearest neighbo$[Title/Abstract]) OR (naive bayes[Title/Abstract]) OR (support vector machine[Title/Abstract])

#6 #4 OR #5

#7 "neoplasms"[Mesh]

#8 (cancer[Title/Abstract]) OR (neoplasm*[Title/Abstract]) OR (tumor*[Title/Abstract])

#9 #7 OR #8

#10 "sensitivity and specificity"[Mesh] OR "area under curve"[Mesh] OR "ROC curve"[Mesh] OR "calibration"[Mesh]

#11 (sensitivity and specificity[Title/Abstract]) OR OR (calibrat*[Title/Abstract]) OR (area under the curve[Title/Abstract]) OR (ROC[Title/Abstract]) OR (AUC[Title/Abstract]) OR (goodness of fit[Title/Abstract]) OR (performance[Title/Abstract]) OR (accuracy*[Title/Abstract])

#12 #10 OR #11

#13 (image*[Title/Abstract])

#14 #3 NOT #6

#15 #9 AND #12 AND #13 AND #14

**2) Embase search strategy：**3506

#1 'artificial intelligence'/exp OR 'machine learning'/exp OR 'deep learning'/exp OR 'artificial neural network'/exp

#2 'decision tree'/exp OR 'random forest'/exp OR 'bayesian learning'/exp OR 'support vector machine'/exp

#3 'malignant neoplasm'/exp OR 'cancer'/exp OR 'tumor*'

#4 'performance'/exp OR 'sensitivity and specificity'/exp OR 'diagnostic accuracy'/exp OR 'area under the curve'/exp OR 'goodness of fit' OR 'calibrat*'

#5 'image*'

#6 #1 NOT #2

#7 #3 AND #4 AND #5 AND #6

**3) IEEE search strategy:** 1409

#1 (Full Text & Metadata:artificial intelligence) OR (Full Text & Metadata:machine learning) OR (Full Text & Metadata:deep learning) OR (Full Text & Metadata:neural network)

#2 (Full Text & Metadata:decision tree) OR (Full Text & Metadata:random forest) OR (Full Text & Metadata:naive bayes) OR (Full Text & Metadata:nearest neighbor) OR (Full Text & Metadata:vector machine)

#3 (Full Text & Metadata:cancer) OR (Full Text & Metadata:neoplasms) OR (Full Text & Metadata:tumor)

#4 (Full Text & Metadata:performance) OR (Full Text & Metadata:sensitivity) OR (Full Text & Metadata:specificity) OR (Full Text & Metadata:accuracy) OR (Full Text & Metadata:area under the curve) OR (Full Text & Metadata:AUC) OR (Full Text & Metadata:ROC) OR (Full Text & Metadata:goodness of fit) OR (Full Text & Metadata:calibrat)

#5 (Full Text & Metadata:image)

#6 #1 NOT #2

#7 #3 AND #4 AND #5 AND #6

**4) Cochrane search strategy:** 572

#1 - (artificial intelligence):ti,ab,kw OR (machine learning):ti,ab,kw OR (deep learning):ti,ab,kw OR (neural network):ti,ab,kw

#2 - (cancer):ti,ab,kw OR (neoplasms):ti,ab,kw OR (tumor):ti,ab,kw

#3 - (accuracy):ti,ab,kw OR (area under curve): ti,ab, kw OR (sensitivity): ti,ab,kw OR (specificity): ti,ab,kw

#4 - #1 AND #2 AND #3

**Supplementary Figure 1: QUADAS-2 summary plot.**

Risk of bias and applicability concerns summary about each QUADAS-2 domain presented as percentages across the 48 included studies.

**
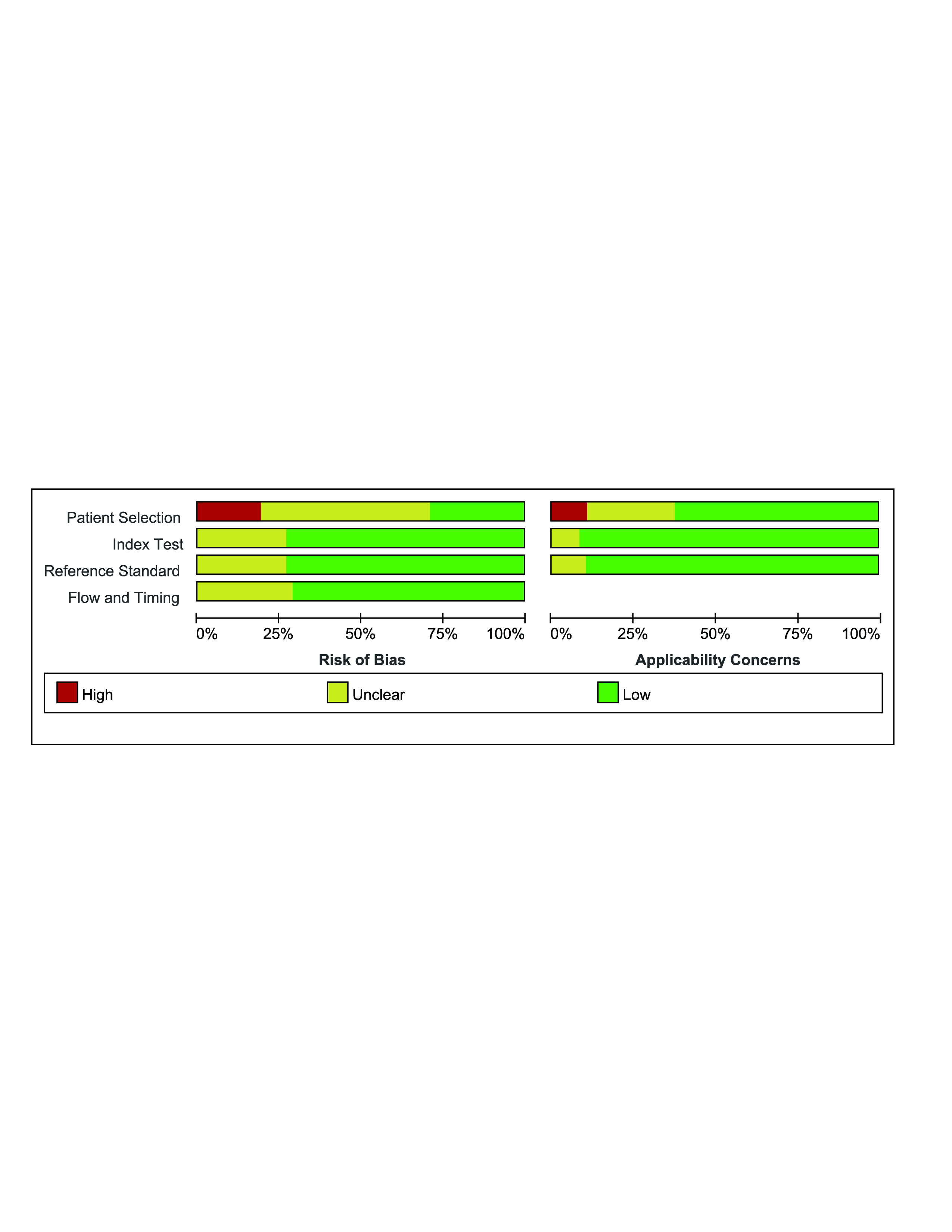
**

**Supplementary Figure 2: Risk of bias and concern of applicability for each item in included studies.**

**
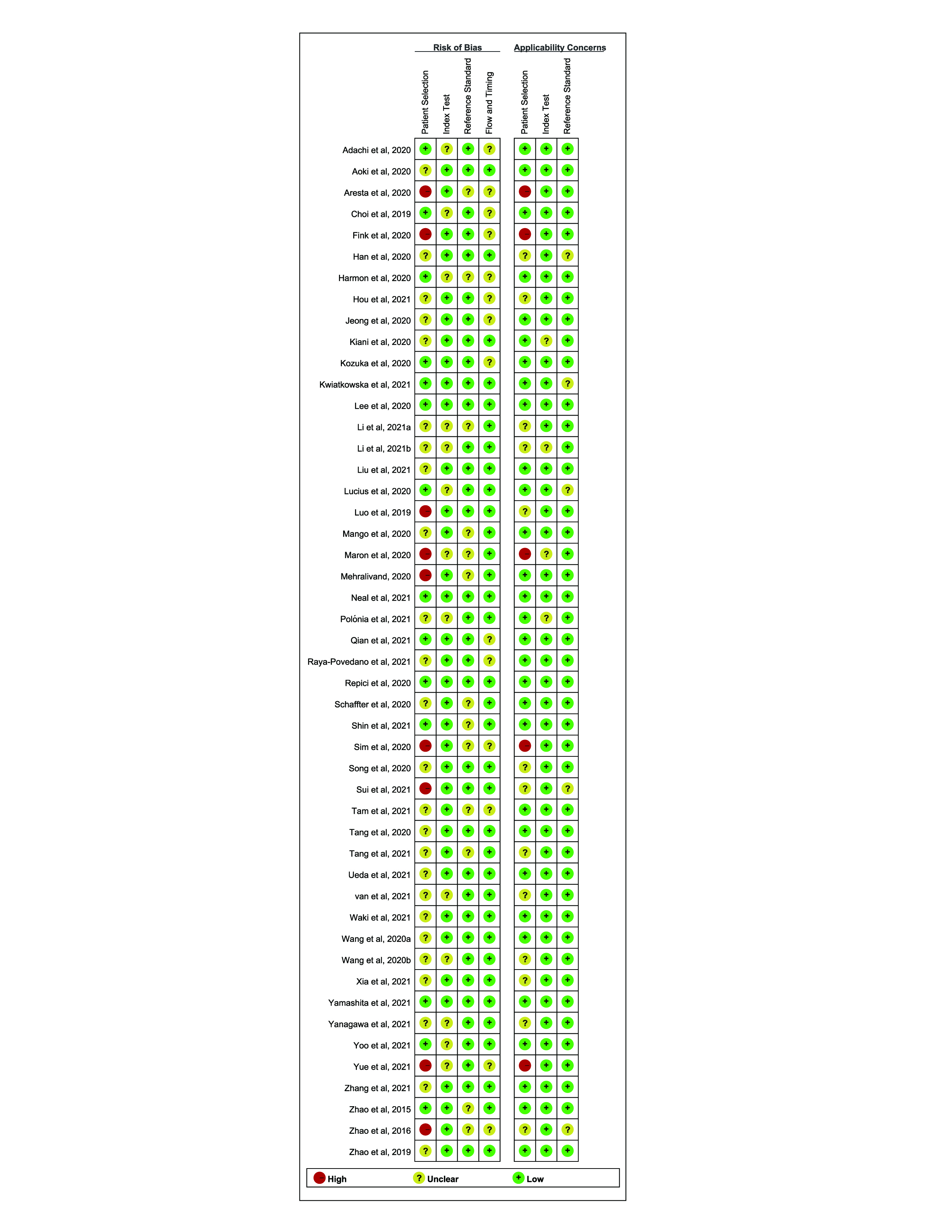
**


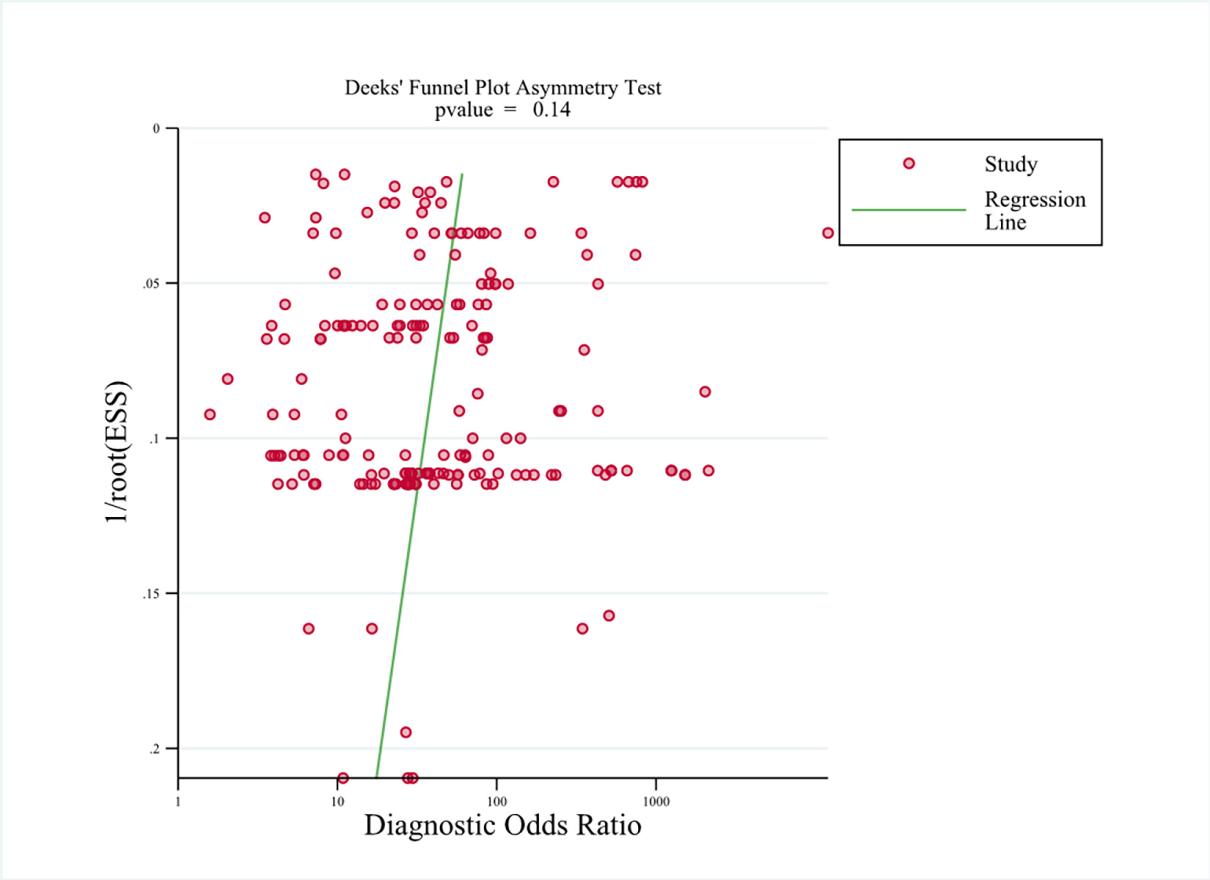
**Supplementary Figure 3: Publication bias.**

Funnel plots suggested there was no publication bias (p = 0.14 > 0.05).

| **Supplementary Table 1: Inclusion/ exclusion criteria** | |
| --- | --- |
| Inclusion criteria | Exclusion criteria |
| - Studies focusing on assessing the performance of clinicians versus DL-assisted clinicians in image-based cancer diagnosis | - Studies of medical waveform data graphics material |
|  | - Studies investigating image segmentation rather than cancer identification |
|  | - Ternary diagnosis outcomes |
|  | - Non-DL studies |
|  | - Case reports, reviews, editorials, letters, comments, conference abstract/proceedings, and duplicates |

| **Supplementary Table 2. Participant demographics** | | | | |
| --- | --- | --- | --- | --- |
| First author and year | Participants | | | |
|  | Inclusion criteria | Exclusion criteria | N | Mean or median age (SD; range) |
| Aoki et al 2020 [1] | Patients with bone scintigraphy confirmed prostate cancer | NR | 139 | 74 (NR; 49-91) |
| Aresta et al 2020 [2] | NR | NR | NR | NR |
| Han et al 2020 [3] | Patients with skin diseases or general skin disorders | Patients with a sign or rare diseases such as ulcer, and purpura | 28,222 | NR |
| Harmon et al 2020 [4] | NR | Missing or low-quality data, or those undergoing more than two types of neoadjuvant therapy | 307 | NR |
| Hou et al 2021 [5] | Patients undergoing radical prostatectomy | Patients with previous surgeries or adjuvant therapies for prostate cancer | 849 | 69·6 (6·9; 42-87) |
| Li et al 2021[6] | NR | NR | 59,214 | NR |
| Polónia et al 2021 [7] | NR | NR | 46 | NR |
| Li et al 2021 [8] | NR | NR | 525 | 47·2 (15·4; 17-91) |
| Neal et al 2021 [9] | NR | Yes | NR | NR |
| Liu et al 2021 [10] | Patients with small bowel malignant tumors | Patients with a history of other primary malignancies; loss of contrast-enhanced CT images; located in the papilla; poor visualization | 87 | 58 (NR; 4-80) |
| Kwiatkowska et al 2021 [11] | NR | NR | NR | NR |
| Kiani et al 2020 [12] | Adult patients with HCC or CC | Patients with combined CC-HCC | 70 | NR |
| Kozuka et al 2020 [13] | Patients (age ≥ 20) with suspected lung cancer | Patients with pneumonia, diffuse lung disease, massive pleural effusion/atelectasis, and severe postoperative complications | 117 | NR |
| Lucius et al 2020 [14] | Patients with melanocytic nevus, vascular skin lesions, benign keratoses, dermatofibroma, intraepithelial carcinoma, basal cell carcinoma and melanoma | NR | NR | NR |
| Mehralivand et al 2020 [15] | Prostate patients and health control with mpMRI | Missing prostatectomy histopathologic lesion maps | 236 | NR |
| Lee et al 2020 [16] | NR | NR | NR | NR |
| Mango et al 2020 [17] | NR | NR | 900 | 53·6 (NR; 17-96) |
| Zhao et al 2019 [18] | Patients who underwent NBI-ME examination | Patients underwent esophagectomy or without IPCLs | 219 | NR |
| Qian et al 2021 [19] | Patients with breast cancer | Mental illness or major underlying diseases; women with implants, pregnancy or lactation; had surgery or chemotherapy | 775 | Development dataset: 46·6 (NR; 23-73); Clinical test dataset: 48·7 (NR; 26-81) |
| Van et al 2021 [20] | NR | Breast implants, poor quality, missing image or truth data | 240 | 56·3 (9·8; 30-81) |
| Yue et al 2021 [21] | Patients with an unspecified infiltrating duct carcinoma | Nonspecific subtype breast cancer with other special types of breast carcinoma; the invasion lesions were not obvious | 50 | NR |
| Tam et al 2021 [22] | NR | Incomplete or corrupted data; tumor mimics | 200 | NR |
| Raya-Povedano et al 2021 [23] | Women who were screened with both two-view digital mammography and two-view digital breast tomosynthesis | Refusal to the study; difficulty retrieving mammograms from the image archiving and communication system | 15,987 | 58 (6; 50-69) |
| Wang et al 2020 [24] | NR | NR | 263 | 51·4 (9·8; 28-76) |
| Xia et al 2021 [25] | Patients with pathological confirmed breast cancer | Poorquality images | 40 | 50·9 (13·9; NR) |
| Repici et al 2020 [26] | 40-80 years old patients underwent colonoscopy and with immunochemical test positivity or symptoms/signs | Patients with history of colorectal cancer, inflammatory bowel disease, previous colonic resection, and antithrombotic therapy precluding polyp resection; lack of informed written consent | 685 | Man: 61·32 (10·2; NR) |
| Maron et al 2020 [27] | NR | NR | NR | NR |
| Schaffter et al 2020 [28] | NR | NR | 153,588 | US: 58·4 (9·7; NR); Swedish: 53·3 (9·4; NR) |
| Sim et al 2019 [29] | Patients with lung cancer nodules needed to be pathologically proven; the number of lesions per radiograph ≤ 3; nodules between 1-3 cm, not in a major airway or mediastinum | Poor quality images | 600 | Healthy adults: 52·4 (12.2; 19-78); Patients with lung cancer: 61·5 (12·3; 20-89) |
| Choi et al 2019 [30] | Women aged ≥ 20 years with breast masses | Women who had masses without definite final diagnoses | 816 | 47 (NR; 42·0-53·5) |
| Adachi et al 2020 [31] | Patients underwent dynamic contrast-enhanced MRI, and patients with confirmed benign or malignant lesions | Patients underwent breast surgery, hormonal therapy, chemotherapy, or radiation therapy; aged ≤ 20 years | 321 | NR |
| Jeong et al 2020 [32] | Patients underwent cholecystectomy and ultrasonography for gallbladder | No polyps found or polyps smaller than 4 mm; polyp was evidently different between ultrasonography and pathologic report; rare pathology | 535 | 52·7 (13·4; 21-87) |
| Fink et al 2020 [33] | Patients with combined naevi and melanomas | NR | 72 | Patients with melanomas: 61·4 (13·1; NR); Patients with naevi: 42·7 (22·5; NR) |
| Zhang et al 2021 [34] | Patients underwent hysteroscopic examination | Poor quality images; images of patients using intrauterine device or receiving hormone therapy; images of patients with multiple uterine diseases; no histopathology was performed | 454 | NR |
| Ueda et al 2021 [35] | Patients surgically diagnosed with lung cancer | Mass lesions >30 mm; metastatic lung cancer; lesions other than pulmonary nodules; ground glass nodules < 5 mm | 312 | 61·6 (11·4; NR) |
| Sui et al 2021 [36] | Patients with pathological confirmed esophageal cancer | Incomplete data or poor-quality images | 514 | Patients with esophageal cancer: 57·4 (NR; 34-87); Negative cancer patients: 41·7 (NR; 18-73) |
| Shin et al 2021 [37] | Patients with glioblastoma or brain metastasis | Multiple enhancing lesions; absent or inadequate MR images; and patients with previous intracranial intervention | 741 | Internal: GBM: 57·3 (15·2; NR); solitary metastases: 57·4 (13·8; NR); External: GBM: 57·2 (12·8; NR); solitary metastases: 61·0 (10·2; NR) |
| Tang et al 2021 [38] | Patients with histologically confirmed malignancies | Patients with multiple synchronous lesions, gastric stump cancer, and missing data | 728 | Development dataset: 63·6 (NR; 31-84); Test dataset: 62·8 (NR; 37-80) |
| Yanagawa et al 2020 [39] | Patients with non-mucinous adenocarcinoma underwent CT examination; no previous treatment; age ≥ 20 years | Patients with histological subtypes other than adenocarcinoma; no preoperative thin-section CT; inadequate data for diagnosis | 285 | 66 (NR; 22-89) |
| Yamashita et al 2020 [40] | Patients underwent primary colorectal cancer resection | NR | 837 | NR |
| Waki et al 2021 [41] | NR | Patients with advanced ESCC and histories of surgery, radiation therapy, and endoscopic treatment for ESCC | 1567 | Validation datasets: 70 (47-88) |
| Yoo et al 2021 [42] | Patients with lung cancer within 1 year of screening | NR | 294 | 62·6 (5·1; NR) |
| Song et al 2020 [43] | NR | NR | 1814 | NR |
| Wang et al 2020 [44] | Patients who underwent thyroid ultrasound imaging and ultrasound-guided FNA with NGS | NR | 249 | 56 (NR; 14) |
| Tang et al 2020 [45] | Patients with EGC; ESD treatment; histologically proven malignancy; and endoscopic examination before ESD | History of chemotherapy or radiation to the stomach, lesions adjacent to the ulcer or ulcer scar, gastric stump cancer, and multiple synchronous cancerous lesions | 1364 | Training dataset: 63·4 (NR; 27-90); Validation dataset: 64·0 (NR; 34-86) |
| Zhao et al 2015 [46] | Patients with complete prostate MR data, underwent ultrasound-guided biopsy, and received long-term follow-up | Patients were previously diagnosed with PCa or prostate sarcoma; there was treatment before the MR scan | 71 | 68·8 (8·9; 40-82) |
| Zhao et al 2016 [47] | NR | NR | NR | NR |
| Luo et al 2019 [48] | Patients with upper gastro intestinal cancer | A history of cancer or gastrointestinal surgery, and undiagnosed upper gastrointestinal disease | 84,424 | NR |
| NR = not reported, MR = magnetic resonance imaging, mpMRI = multiparametric magnetic MRI, MR = magnetic resonance, HCC=hepatocellular carcinoma, CC = cholangiocarcinoma, NBI = narrow-band imaging, NBI-ME = the combination of NBI and magnifying endoscopy, IPCLs = intrapapillary capillary loops, GBM = glioblastoma, WHO = World Health Organization, ESCC = esophageal squamous cell carcinoma, FNA = fine needle aspiration, NGS = next-generation sequencing, EGC = early gastric cancer, ESD = endoscopic submucosal dissection. | | | | |
|  |  |  |  |  |
|  |  |  |  |  |

| **Supplementary Table 3. Model training and validation** | | | | |
| --- | --- | --- | --- | --- |
| First author and year | Target condition | Reference standard | Type of internal validation | External validation |
| Aoki et al 2020 [1] | Endocrine cancer | Medical record, at least one-year follow-up | NR | No |
| Aresta et al 2020 [2] | Lung cancer | Expert consensus | NR | No |
| Han et al 2020 [3] | Others (skin cancer) | Histopathology or expert consensus | NR | Yes |
| Harmon et al 2020 [4] | Others (bladder cancer) | Expert consensus | Random split-sample validation | Yes |
| Hou et al 2021 [5] | Endocrine cancer | Histopathology | Random split-sample validation | Yes |
| Li et al 2021[6] | Breast cancer | Histopathology | NR | Yes |
| Polónia et al 2021 [7] | Breast cancer | Histopathology | NR | Yes |
| Li et al 2021 [8] | Breast cancer | Histopathology | Four-fold cross-validation | Yes |
| Neal et al 2021 [9] | Lung cancer | Expert consensus | Ten-fold cross-validation | No |
| Liu et al 2021 [10] | Others (lymphoma) | Histopathology | Cross-validation | No |
| Kwiatkowska et al 2021 [11] | Others (skin cancer) | Histopathology | Random split-sample validation | No |
| Kiani et al 2020 [12] | Gastrointestinal cancer | Histopathology or expert consensus | Random split-sample validation | Yes |
| Kozuka et al 2020 [13] | Lung cancer | Expert consensus | NR | No |
| Lucius et al 2020 [14] | Others (skin cancer) | Expert consensus | Random split-sample validation | No |
| Mehralivand et al 2020 [15] | Endocrine cancer | Histopathology | NR | No |
| Lee et al 2020 [16] | Endocrine cancer | Histopathology or expert consensus | Random split-sample validation | Yes |
| Mango et al 2020 [17] | Breast cancer | Histopathology | NR | Yes |
| Zhao et al 2019 [18] | Gastrointestinal cancer | Histopathology | Three-fold cross-validation | No |
| Qian et al 2021 [19] | Breast cancer | Histopathology | Random split-sample validation | Yes |
| Van et al 2021 [20] | Breast cancer | Histopathology | NR | No |
| Yue et al 2021 [21] | Breast cancer | Expert consensus | NR | Yes |
| Tam et al 2021 [22] | Lung cancer | Expert consensus | NR | No |
| Raya-Povedano et al 2021 [23] | Breast cancer | Histopathology | NR | Yes |
| Wang et al 2020 [24] | Breast cancer | Histopathology, 2 years follow-up | Five-fold cross-validation | No |
| Xia et al 2021 [25] | Breast cancer | Histopathology | NR | No |
| Repici et al 2020 [26] | Gastrointestinal cancer | Histopathology | NR | Yes |
| Maron et al 2020 [27] | Others (skin cancer) | Histopathology | NR | No |
| Schaffter et al 2020 [28] | Breast Cancer | Histopathology | Ten-fold cross-validation | Yes |
| Sim et al 2019 [29] | Lung Cancer | Histopathology | NR | Yes |
| Choi et al 2019 [30] | Breast cancer | Histopathology | NR | No |
| Adachi et al 2020 [31] | Breast cancer | Histopathology | Random split-sample validation | No |
| Jeong et al 2020 [32] | Gastrointestinal cancer | Histopathology | NR | Yes |
| Fink et al 2020 [33] | Others (skin cancer) | Histopathology or expert consensus | NR | Yes |
| Zhang et al 2021 [34] | Other (endometrial cancer) | Histopathology | Random split-sample validation | No |
| Ueda et al 2021 [35] | Lung cancer | Histopathology | NR | Yes |
| Sui et al 2021 [36] | Gastrointestinal cancer | Histopathology | Random split-sample validation | Yes |
| Shin et al 2021 [37] | Endocrine cancer | Histopathology | Random split-sample validation | Yes |
| Tang et al 2021 [38] | Gastrointestinal cancer | Histopathology | Random split-sample validation | Yes |
| Yanagawa et al 2020 [39] | Lung cancer | Histopathology | Ten-fold cross-validation | No |
| Yamashita et al 2020 [40] | Gastrointestinal cancer | Histopathology | Four-fold cross-validation | Yes |
| Waki et al 2021 [41] | Gastrointestinal cancer | Histopathology | NR | Yes |
| Yoo et al 2021 [42] | Lung cancer | Histopathology | NR | No |
| Song et al 2020 [43] | Gastrointestinal cancer | Histopathology | Random split-sample validation | Yes |
| Wang et al 2020 [44] | Endocrine cancer | Histopathology | Random split-sample validation | No |
| Tang et al 2020 [45] | Gastrointestinal cancer | Histopathology | Random split-sample validation | Yes |
| Zhao et al 2015 [46] | Endocrine cancer | Histopathology with or without a long-term follow-up | Cross-validation | No |
| Zhao et al 2016 [47] | Others (brain tumor) | Expert consensus | NR | No |
| Luo et al 2019 [48] | Gastrointestinal cancer | Histopathology | Random split-sample validation | Yes |

| **Supplementary Table 4. Indicators, algorithms and data sources** | | | | | | | | | | |
| --- | --- | --- | --- | --- | --- | --- | --- | --- | --- | --- |
| First author and year | Indicator definition | | | Algorithm | | | | Data source | | |
|  | Device | EPQI | Heatmap provided | Algorithm name | Transfer learning | Source of data | SSC | Number of images for training/internal/external | Data range | Open access data |
| Aoki et al 2020 [1] | Others (scintigraphy) | NR | No | BtrflyNets | No | Retrospective study, data from department of Radiology, Asahikawa Medical University, Hokkaido, Japan | NR | NR/NR/NR | 2009-2018 | No |
| Aresta et al 2020 [2] | Others (CT) | NR | No | CNN | Yes | Retrospective study, data from a publicly available dataset | NR | NR/NR/NR | NR | Yes |
| Han et al 2020 [3] | Others (dermoscopy) | NR | No | CNN | No | Retrospective study, training from Seoul National University Bundang Hospital, validation from Edinburgh commercially available images and Seoul National University Bundang Hospital, Inje University Sanggye Paik Hospital, and Hallym University Dongtan Hospital | NR | 220680/NR/3501 | NR | Yes |
| Harmon et al 2020 [4] | Others (WSI) | Yes | No | DL | No | Retrospective study, data from National Cancer Institute Genomic Data Commons Data Portal and author's local institution | NR | 219/73/89 | NR | Yes |
| Hou et al 2021 [5] | MRI | NR | No | ResNet | No | Retrospective study, data from two care medical centers | NR | 596/150/103 | 2015-2019 | No |
| Li et al 2021[6] | X-ray | NR | No | CNN, ResNet | No | Retrospective study, training and validation across six cities of China | NR | 58516/NR/608 | 2015-2019 | No |
| Polónia et al 2021 [7] | Others (WSI) | NR | No | CNN | No | Retrospective study, data from two histology laboratories | NR | NR/NR/252 | 2013-2017 | No |
| Li et al 2021 [8] | Ultrasound | NR | No | DenseNet | No | Retrospective study, data from Shanghai General Hospital | NR | NR/NR/599 | 2018-2019 | No |
| Neal et al 2021 [9] | Others (CT) | Yes | Yes | CNN | No | Retrospective study, data from LIDC/IDRI and LUNA 16 | NR | 1400/600/NR | NR | Yes |
| Liu et al 2021 [10] | Others (CT) | Yes | No | DL | No | Retrospective study, data from the Affiliated hospital of Qingdao | NR | 87/NR/NR | 2013-2019 | No |
| Kwiatkowska et al 2021 [11] | Others (dermoscopy) | NR | Yes | CNN | Yes | Retrospective study, data from ISIC 2018: Skin Lesion Analysis Towards Melanoma Detection grand challenge | NR | 8123/886/NR | NR | Yes |
| Kiani et al 2020 [12] | Others (WSI) | Yes | Yes | DenseNet | No | Retrospective study, data from Stanford University Medical Center | NR | 22400/2600/80 | 2011-2017 | Yes |
| Kozuka et al 2020 [13] | Others (CT) | Yes | No | Faster R-CNN | No | Retrospective study, data from Kindai University Hospital | NR | 117/NR/NR | 2018.11-12 | Yes |
| Lucius et al 2020 [14] | Others (dermoscopy) | NR | No | DNN | Yes | Retrospective study, data from ISIC archive | NR | 8313/1702/NR | NR | Yes |
| Mehralivand et al 2020 [15] | MRI | NR | No | DL | No | Retrospective study, data from five institutions | NR | 161/NR/NR | NR | No |
| Lee et al 2020 [16] | Others (CT) | NR | Yes | CNN | No | Retrospective study | NR | 787/104/3838 | 2018.01-08 | No |
| Mango et al 2020 [17] | Ultrasound | NR | No | ANN | No | Retrospective study, data from over 21 US institutions | NR | NR/NR/900 | 2004-2016 | No |
| Zhao et al 2019 [18] | Endoscopy | NR | No | FCN, CNN | Yes | Retrospective study, data from First Affiliated Hospital of Anhui Medical University of China | NR | NR/NR/NR | 2015.03-2017.02 | No |
| Qian et al 2021 [19] | Ultrasound | Yes | Yes | ResNet-18 | Yes | Prospective study, data from Chinese Hospitals | NR | NR/NR/912 | 2016-2018 | No |
| Van et al 2021 [20] | X-ray | Yes | No | DCNN | No | Retrospective study, data from seven US clinical sites | Yes | 8640/NR/NR | 2011-2014 | No |
| Yue et al 2021 [21] | Other (WSI) | Yes | Yes | FCN | No | Retrospective study, data from the Fourth Hospital of Hebei Medical University of China | NR | NR/NR/50 | 2019.01-12 | No |
| Tam et al 2021 [22] | X-ray | NR | Yes | DCNN | No | Retrospective study, data from the NHS Cancer Registry | NR | 396/NR/NR | NR | Yes |
| Raya-Povedano et al 2021 [23] | X-ray | NR | No | DL | No | Retrospective study, data from Córdoba Tomosynthesis Screening Trial | NR | NR/NR/15987 | 2015.01-2016.12 | No |
| Wang et al 2020 [24] | Ultrasound | NR | No | CNN | Yes | Retrospective study, data from Jeonbuk National University Hospital | NR | 316/316/NR | 2012-2018 | No |
| Xia et al 2021 [25] | Ultrasound | NR | Yes | CNN, Inception-v3 | No | Retrospective study, data from Anqing First People’s Hospital Affiliated to Anhui Medical University | NR | 40/NR/NR | 2019.11-2020.06 | No |
| Repici et al 2020 [26] | Others (colonoscopy) | NR | No | CNN | No | Retrospective study, data were obtained from at three centers in Italy | Yes | NR/NR/685 | 2019.09-11 | No |
| Maron et al 2020 [27] | Others (dermoscopy) | NR | No | CNN | No | Retrospective study, images were obtained from the ISIC archive | NR | 4894/4894/NR | 2019.06-08 | Yes |
| Schaffter et al 2020 [28] | X-ray | NR | No | Faster R-CNN | No | Retrospective study, data from Kaiser Permanente Washington and the Karolinska Institute | NR | 100974/43257/166578 | 2008.04-2012.12 | No |
| Sim et al 2019 [29] | X-ray | NR | No | DCNN | No | Retrospective study, data from four centers, Freiburg University Hospital, Massachusetts General Hospital, Samsung Medical Center, and Severance Hospital | Yes | 17210/2120/800 | 2015.10-2017.09 | No |
| Choi et al 2019 [30] | Ultrasound | NR | No | DL | No | Retrospective study, data from Samsung Medical Center | NR | 253/NR/NR | 2015.01-12 | No |
| Adachi et al 2020 [31] | MRI | NR | No | RetinaNet | No | Retrospective study, data from Tokyo Medical and Dental University Hospital | NR | 184/72/NR | 2014.03- 2018.10 | No |
| Jeong et al 2020 [32] | Ultrasound | Yes | Yes | CNN | Yes | Retrospective study, data from Seoul National University Hospital | NR | 5171/NR/885 | 2006-2017 | No |
| Fink et al 2020 [33] | Others (dermoscopy) | Yes | Yes | CNN | No | Retrospective study, data from the university medical centers of Heidelberg, and the medical center, Munich | NR | NR/NR/72 | NR | No |
| Zhang et al 2021 [34] | Others (Hysteroscopy) | Yes | Yes | VGGNet-16, CNN | Yes | Retrospective study, data from the Shengjing Hospital of China Medical University | NR | 6478/250/NR | 2017-2019 | No |
| Ueda et al 2021 [35] | X-ray | NR | No | DL | No | Retrospective study, data from Osaka City University Hospital | NR | NR/NR/312 | 2017-2018 | No |
| Sui et al 2021 [36] | Others (CT) | Yes | No | CNN | No | Retrospective study, data from the China-Japan Union Hospital of Jilin University | NR | 222/92/100 | 2017.02-2019.04 | No |
| Shin et al 2021 [37] | MRI | NR | Yes | ResNet50 | No | Retrospective study, data from National Health Insurance Corporation Ilsan Hospital | NR | 450/100/143 | 2006.02-2017.12 | No |
| Tang et al 2021 [38] | Endoscopy | Yes | Yes | DCNN | No | Retrospective study, data from two institutions of China | NR | 3407/3407/228 | 2017-2019 | No |
| Yanagawa et al 2020 [39] | Others (CT) | Yes | No | 3D-CNN | No | Retrospective study, data from three institutions of Japan | NR | NR/NR/NR | 2009.01-2011.12 | No |
| Yamashita et al 2020 [40] | Others (WSI) | NR | Yes | DL | Yes | Retrospective study, data from Stanford University Medical Center | NR | 75/15/484 | 2015.01-2017.12 | Yes |
| Waki et al 2021 [41] | Endoscopy | NR | No | DL | No | Retrospective study, data from Osaka International Cancer Institute, Fukuoka University Chikushi Hospital, and Niigata University Hospital | Yes | 18797/NR/100 | 2005.12-2019.06 | No |
| Yoo et al 2021 [42] | X-ray | NR | Yes | Resnet | No | Retrospective study, data from NLST data | NR | 519/NR/NR | 2002-2004 | Yes |
| Song et al 2020 [43] | Others (WSI) | NR | Yes | CNN | No | Retrospective study, data from multicenter hospitals of China | NR | 2123/100/1582 | 2017.06-08 | No |
| Wang et al 2020 [44] | Ultrasound | NR | No | Auto ML | Yes | Retrospective study, data from Thomas Jefferson University Hospital | NR | 716/51/NR | 2017-2019 | No |
| Tang et al 2020 [45] | Endoscopy | Yes | No | DCNN | No | Retrospective study, data from four institutions in China | NR | 35823/9417/10931 | 2016-2019 | No |
| Zhao et al 2015 [46] | MRI | NR | No | ANN | No | Retrospective study, data from prostate MR database | NR | 426/426/NR | 2008-2010 | Yes |
| Zhao et al 2016 [47] | MRI | NR | No | CNN | No | Retrospective study, data from Multimodal Brain Tumor Image Segmentation Benchmark | NR | NR/NR/NR | NR | Yes |
| Luo et al 2019 [48] | Endoscopy | Yes | Yes | DL | No | Retrospective study, data from six hospitals across China | NR | 125898/15637/812539 | NR | No |
| Exclusion of poor quality imaging = EPQI, Sample size calculation = SSC, NR = not reported, MRI = magnetic resonance imaging, mpMRI = multi-parametric MRI, WSI = whole-slide image, DL = deep learning, BtrflyNets = butterfly-type networks, CNN = convolutional neural network, YOLO = you only look once, ResNet = deep residual network, ISIC = International Skin Imaging Collaboration, Auto ML = automated machine learning, DCNN = deep CNN, ANN = artificial neural network, 3D-CNN = three dimensional-CNN, VGGNet = visual geometry group network, R-CNN = region with CNN feature, FCN = fully convolutional network, DenseNet = dense convolutional network. | | | | | | | | | | |

**Supplementary Table 5: Meta-regression result**

| Covariates | Subgroup | LRTChi2 | I^2^ | *P* value |
| --- | --- | --- | --- | --- |
| Cancer type | Breast cancer | 2.46 | 19 | 0.29 |
|  | Lung cancer |  |  |  |
|  | Gastrointestinal cancer |  |  |  |
|  | Endocrine cancer |  |  |  |
|  | Others |  |  |  |
| Device type | Ultrasound | 7.97 | 75 | 0.02 |
|  | X-ray |  |  |  |
|  | Endoscopy |  |  |  |
|  | MRI |  |  |  |
|  | Others |  |  |  |

**References (Full list of included 48 studies for systematic review (*25 studies for** **meta-analysis)**

1. *Aoki Y, Nakayama M, Nomura K, et al. The utility of a deep learning-based algorithm for bone scintigraphy in patient with prostate cancer. Ann Nucl Med 2020; 34(12): 926-31.
2. Aresta G, Ferreira C, Pedrosa J, et al. Automatic Lung Nodule Detection Combined with Gaze Information Improves Radiologists' Screening Performance. IEEE J Biomed Health Inform 2020; 24(10): 2894-901.
3. Han SS, Park I, Eun Chang S, et al. Augmented Intelligence Dermatology: Deep Neural Networks Empower Medical Professionals in Diagnosing Skin Cancer and Predicting Treatment Options for 134 Skin Disorders. J Invest Dermatol 2020; 140(9): 1753-61.
4. Harmon SA, Sanford TH, Brown GT, et al. Multiresolution Application of Artificial Intelligence in Digital Pathology for Prediction of Positive Lymph Nodes from Primary Tumors in Bladder Cancer. JCO Clin Cancer Inform 2020; 4: 367-82.
5. *Hou Y, Zhang YH, Bao J, et al. Artificial intelligence is a promising prospect for the detection of prostate cancer extracapsular extension with mpMRI: a two-center comparative study. Eur J Nucl Med Mol Imaging 2021; 48(12): 3805-16.
6. Li H, Ye J, Liu H, et al. Application of deep learning in the detection of breast lesions with four different breast densities. Cancer Med 2021; 10(14): 4994-5000.
7. Polónia A, Campelos S, Ribeiro A, et al. Artificial Intelligence Improves the Accuracy in Histologic Classification of Breast Lesions. Am J Clin Pathol 2021; 155(4): 527-36.
8. *Li C, Li J, Tan T, Chen K, Xu Y, Wu R. Application of ultrasonic dual-mode artificially intelligent architecture in assisting radiologists with different diagnostic levels on breast masses classification. Diagn Interv Radiol 2021; 27(3): 315-22.
9. Neal Joshua ES, Bhattacharyya D, Chakkravarthy M, Byun YC. 3D CNN with Visual Insights for Early Detection of Lung Cancer Using Gradient-Weighted Class Activation. J Healthc Eng 2021; 2021: 6695518.
10. Liu S, Zhang C, Liu R, et al. CT Texture Analysis for Preoperative Identification of Lymphoma from Other Types of Primary Small Bowel Malignancies. Biomed Res Int 2021; 2021: 5519144.
11. Kwiatkowska D, Kluska P, Reich A. Convolutional neural networks for the detection of malignant melanoma in dermoscopy images. Postepy Dermatol Alergol 2021; 38(3): 412-20.
12. *Kiani A, Uyumazturk B, Rajpurkar P, et al. Impact of a deep learning assistant on the histopathologic classification of liver cancer. NPJ Digit Med 2020; 3: 23.
13. *Kozuka T, Matsukubo Y, Kadoba T, et al. Efficiency of a computer-aided diagnosis (CAD) system with deep learning in detection of pulmonary nodules on 1-mm-thick images of computed tomography. Jpn J Radiol 2020; 38(11): 1052-61.
14. Lucius M, De All J, De All JA, et al. Deep Neural Frameworks Improve the Accuracy of General Practitioners in the Classification of Pigmented Skin Lesions. Diagnostics (Basel) 2020; 10(11).
15. *Mehralivand S, Harmon SA, Shih JH, et al. Multicenter Multireader Evaluation of an Artificial Intelligence-Based Attention Mapping System for the Detection of Prostate Cancer with Multiparametric MRI. AJR Am J Roentgenol 2020; 215(4): 903-12.
16. *Lee JH, Ha EJ, Kim D, et al. Application of deep learning to the diagnosis of cervical lymph node metastasis from thyroid cancer with CT: external validation and clinical utility for resident training. Eur Radiol 2020; 30(6): 3066-72.
17. Mango VL, Sun M, Wynn RT, Ha R. Should We Ignore, Follow, or Biopsy? Impact of Artificial Intelligence Decision Support on Breast Ultrasound Lesion Assessment. AJR Am J Roentgenol 2020; 214(6): 1445-52.
18. Zhao YY, Xue DX, Wang YL, et al. Computer-assisted diagnosis of early esophageal squamous cell carcinoma using narrow-band imaging magnifying endoscopy. Endoscopy 2019; 51(4): 333-41.
19. Qian X, Pei J, Zheng H, et al. Prospective assessment of breast cancer risk from multimodal multiview ultrasound images via clinically applicable deep learning. Nat Biomed Eng 2021; 5(6): 522-32.
20. van Winkel SL, Rodríguez-Ruiz A, Appelman L, et al. Impact of artificial intelligence support on accuracy and reading time in breast tomosynthesis image interpretation: a multi-reader multi-case study. Eur Radiol 2021; 31(11): 8682-91.
21. Yue M, Zhang J, Wang X, et al. Can AI-assisted microscope facilitate breast HER2 interpretation? A multi-institutional ring study. Virchows Arch 2021; 479(3): 443-9.
22. *Tam M, Dyer T, Dissez G, et al. Augmenting lung cancer diagnosis on chest radiographs: positioning artificial intelligence to improve radiologist performance. Clin Radiol 2021; 76(8): 607-14.
23. Raya-Povedano JL, Romero-Martín S, Elías-Cabot E, Gubern-Mérida A, Rodríguez-Ruiz A, Álvarez-Benito M. AI-based Strategies to Reduce Workload in Breast Cancer Screening with Mammography and Tomosynthesis: A Retrospective Evaluation. Radiology 2021; 300(1): 57-65.
24. *Wang Y, Choi EJ, Choi Y, Zhang H, Jin GY, Ko SB. Breast Cancer Classification in Automated Breast Ultrasound Using Multiview Convolutional Neural Network with Transfer Learning. Ultrasound Med Biol 2020; 46(5): 1119-32.
25. *Xia Q, Cheng Y, Hu J, et al. Differential diagnosis of breast cancer assisted by S-Detect artificial intelligence system. Math Biosci Eng 2021; 18(4): 3680-9.
26. Repici A, Badalamenti M, Maselli R, et al. Efficacy of Real-Time Computer-Aided Detection of Colorectal Neoplasia in a Randomized Trial. Gastroenterology 2020; 159(2): 512-20.e7.
27. *Maron RC, Utikal JS, Hekler A, et al. Artificial Intelligence and Its Effect on Dermatologists' Accuracy in Dermoscopic Melanoma Image Classification: Web-Based Survey Study. J Med Internet Res 2020; 22(9): e18091.
28. Schaffter T, Buist DSM, Lee CI, et al. Evaluation of Combined Artificial Intelligence and Radiologist Assessment to Interpret Screening Mammograms. JAMA Netw Open 2020; 3(3): e200265.
29. *Sim Y, Chung MJ, Kotter E, et al. Deep Convolutional Neural Network-based Software Improves Radiologist Detection of Malignant Lung Nodules on Chest Radiographs. Radiology 2020; 294(1): 199-209.
30. *Choi JS, Han BK, Ko ES, et al. Effect of a Deep Learning Framework-Based Computer-Aided Diagnosis System on the Diagnostic Performance of Radiologists in Differentiating between Malignant and Benign Masses on Breast Ultrasonography. Korean J Radiol 2019; 20(5): 749-58.
31. *Adachi M, Fujioka T, Mori M, et al. Detection and Diagnosis of Breast Cancer Using Artificial Intelligence Based assessment of Maximum Intensity Projection Dynamic Contrast-Enhanced Magnetic Resonance Images. Diagnostics (Basel) 2020; 10(5).
32. *Jeong Y, Kim JH, Chae HD, et al. Deep learning-based decision support system for the diagnosis of neoplastic gallbladder polyps on ultrasonography: Preliminary results. Sci Rep 2020; 10(1): 7700.
33. Fink C, Blum A, Buhl T, et al. Diagnostic performance of a deep learning convolutional neural network in the differentiation of combined naevi and melanomas. J Eur Acad Dermatol Venereol 2020; 34(6): 1355-61.
34. Zhang Y, Wang Z, Zhang J, et al. Deep learning model for classifying endometrial lesions. J Transl Med 2021; 19(1): 10.
35. *Ueda D, Yamamoto A, Shimazaki A, et al. Artificial intelligence-supported lung cancer detection by multi-institutional readers with multi-vendor chest radiographs: a retrospective clinical validation study. BMC Cancer 2021; 21(1): 1120.
36. Sui H, Ma R, Liu L, Gao Y, Zhang W, Mo Z. Detection of Incidental Esophageal Cancers on Chest CT by Deep Learning. Front Oncol 2021; 11: 700210.
37. *Shin I, Kim H, Ahn SS, et al. Development and Validation of a Deep Learning-Based Model to Distinguish Glioblastoma from Solitary Brain Metastasis Using Conventional MR Images. AJNR Am J Neuroradiol 2021; 42(5): 838-44.
38. *Tang D, Zhou J, Wang L, et al. A Novel Model Based on Deep Convolutional Neural Network Improves Diagnostic Accuracy of Intramucosal Gastric Cancer (With Video). Front Oncol 2021; 11: 622827.
39. *Yanagawa M, Niioka H, Kusumoto M, et al. Diagnostic performance for pulmonary adenocarcinoma on CT: comparison of radiologists with and without three-dimensional convolutional neural network. Eur Radiol 2021; 31(4): 1978-86.
40. Yamashita R, Long J, Longacre T, et al. Deep learning model for the prediction of microsatellite instability in colorectal cancer: a diagnostic study. Lancet Oncol 2021; 22(1): 132-41.
41. *Waki K, Ishihara R, Kato Y, et al. Usefulness of an artificial intelligence system for the detection of esophageal squamous cell carcinoma evaluated with videos simulating overlooking situation. Dig Endosc 2021; 33(7): 1101-9.
42. *Yoo H, Lee SH, Arru CD, et al. AI-based improvement in lung cancer detection on chest radiographs: results of a multi-reader study in NLST dataset. Eur Radiol 2021; 31(12): 9664-74.
43. *Song Z, Zou S, Zhou W, et al. Clinically applicable histopathological diagnosis system for gastric cancer detection using deep learning. Nat Commun 2020; 11(1): 4294.
44. *Wang F, Liu X, Yuan N, et al. Study on automatic detection and classification of breast nodule using deep convolutional neural network system. J Thorac Dis 2020; 12(9): 4690-701.
45. *Tang D, Wang L, Ling T, et al. Development and validation of a real-time artificial intelligence-assisted system for detecting early gastric cancer: A multicentre retrospective diagnostic study. EBioMedicine 2020; 62: 103146.
46. Zhao K, Wang C, Hu J, et al. Prostate cancer identification: quantitative analysis of T2-weighted MR images based on a back propagation artificial neural network model. Sci China Life Sci 2015; 58(7): 666-73.
47. Zhao L, Jia K. Multiscale CNNs for Brain Tumor Segmentation and Diagnosis. Comput Math Methods Med 2016; 2016: 8356294.
48. *Luo H, Xu G, Li C, et al. Real-time artificial intelligence for detection of upper gastrointestinal cancer by endoscopy: a multicentre, case-control, diagnostic study. Lancet Oncol 2019; 20(12): 1645-54.
